# Respiratory Disease Detection: A Systematic Review of AI-Based Approaches, from Audio and Visual Unimodal Methods to Multimodal Integration

**DOI:** 10.3390/diagnostics16121890

**Published:** 2026-06-17

**Authors:** Asmaa Shati, Ahmed Abdulmutaali, Norah Alsaeed

**Affiliations:** 1 Informatics and Computer Systems Department, King Khalid University, Abha 61421, Saudi Arabia; 2 Department of Chemical Engineering, King Khalid University, Abha 61421, Saudi Arabia

**Keywords:** respiratory diseases, signal processing, medical imaging, machine learning, deep learning

## Abstract

**Background:** Respiratory diseases (RDs), including asthma, COVID-19, chronic obstructive pulmonary disease (COPD), and pneumonia, remain a major global health challenge, contributing substantially to global morbidity and mortality. Conventional diagnosis relies heavily on clinicians’ expertise to interpret respiratory sounds and radiographic images, a process that can be subjective, time-consuming, and prone to inter-observer variability. Recent advances in artificial intelligence (AI) and machine learning (ML) have enabled automated diagnostic approaches that can improve the efficiency, consistency, and scalability of respiratory disease detection. However, existing research remains fragmented across different data modalities. **Methods:** This review systematically analyzes recent studies on AI-based respiratory disease detection using both visual modalities (e.g., chest X-rays, computed tomography (CT) scans, and ultrasound) and audio modalities (e.g., cough and breath sounds). To provide a comprehensive perspective, the reviewed literature is organized using a unified taxonomy that categorizes existing approaches into three main groups: audio-based, visual-based, and audio–visual-based methods. In addition, two conceptual frameworks are proposed to illustrate representative pipelines for audio-based and visual-based respiratory disease classification. **Results:** The analysis reveals that most existing studies focus on single-modality approaches, while multimodal integration remains relatively underexplored. Only a limited number of studies combine audio and visual data within unified frameworks, primarily due to the scarcity of synchronized multimodal datasets collected from the same patients. The proposed taxonomy and conceptual frameworks provide a structured basis for comparing existing methods, identifying methodological trends, and highlighting key research gaps in multimodal respiratory disease detection. **Conclusions:** Future research should prioritize the development of multimodal datasets, robust evaluation protocols, and interpretable and lightweight AI models suitable for real-world clinical deployment. Advancing multimodal integration has the potential to significantly enhance the accuracy, reliability, and clinical applicability of AI-driven respiratory disease diagnosis systems.

## 1. Introduction

Respiratory diseases (RDs), including the diagnosis and illness of COVID-19, tuberculosis (TB), and pneumonia, represent a significant global health burden, collectively accounting for millions of diagnoses, illnesses, and deaths annually [1]. As of 8 February 2026, the World Health Organization reports 7,109,667 global deaths due to COVID-19, underscoring the pandemic’s continued impact on public health [2]. Similarly, the latest Global Tuberculosis Report, released in November 2024, revealed that approximately 8.2 million people were diagnosed with TB, marking the highest number recorded since global TB monitoring began in 1995 [3,4]. Furthermore, pneumonia remains a leading cause of death among children under five, accounting for 14% of all fatalities in this age group, with 740,180 deaths reported globally [5]. These statistics emphasize the urgent need for effective preventive measures and treatment strategies to address the critical public health challenges posed by respiratory diseases.

The diagnosis of respiratory conditions primarily relies on two main approaches: clinical methods and computer-based techniques [6]. Clinical methods encompass three categories: traditional general examination methods, history-based evaluations, and histopathological image analysis [6]. On the other hand, computer-based techniques utilize audio and visual modalities to process respiratory sounds and images for diagnostic purposes.

Audio and visual modalities are pivotal in diagnosing and managing respiratory diseases, with the integration of machine learning (ML) and deep learning (DL) significantly enhancing their effectiveness. Audio-based modalities, such as cough analysis, can help identify conditions such as asthma, pneumonia, or COVID-19 through distinct acoustic features [7,8]. Additionally, wheezes—high-pitched respiratory sounds indicative of conditions like asthma and COPD—and crackles, often associated with pulmonary fibrosis or pneumonia, can be effectively diagnosed through lung sound recordings analyzed with advanced deep learning methods [9,10]. On the other hand, visual modalities employ imaging techniques such as chest X-rays (CXR), which are essential for detecting structural abnormalities like consolidations or fluid buildup, and Computed Tomography (CT) scans, which offer detailed cross-sectional views of the lungs to identify nodules, emphysema, or interstitial lung diseases [11,12]. By leveraging ML and DL models trained on these acoustic and imaging modalities, researchers have achieved high accuracy in detecting and classifying lung diseases, thereby significantly enhancing diagnostic capabilities [7,13].

ML is a promising approach with the potential to effectively utilize medical data, enabling significant improvements in healthcare quality and cost efficiency [8,14]. Datasets play a crucial role in developing ML and DL models for diagnosing and managing respiratory diseases, serving as the primary resource for training and validation. The selection of an appropriate dataset is critical for ensuring the generalizability and accuracy of models across diverse clinical applications [15]. Moreover, the availability of datasets—whether publicly accessible or restricted—has a profound impact on the scope and advancement of research efforts. Publicly accessible datasets facilitate open collaboration and consistent benchmarking, thus fostering innovation and ensuring reproducibility in studies. Conversely, private datasets, while often richer in detail, impose barriers to widespread research and limit opportunities for broader collaboration. Remarkable efforts have been made by researchers to make datasets publicly available. Notable examples include the release of the NIH chest X-ray database [16] and the ICBHI 2017 Challenge database [17], which have become invaluable resources for the research community.

Several reviews have been conducted on the detection of RD using audio or visual modalities using ML and DL techniques [6,11,18,19,20]. Reviews in [11,18,19] focus on studies based on the methods employed and the types of lung diseases identified through visual modalities such as CXR and CT scans. Other reviews [6,20] provide insights into lung disease detection using acoustic signal analysis, including coughs, wheezes, crackles, and diagnostics from voice and speech, leveraging ML and DL techniques. Collectively, these reviews highlight the growing importance of advanced methods in improving the detection and diagnosis of RD through diverse modalities.

Although significant progress has been made in detecting RDs, a comprehensive survey of state-of-the-art approaches across both audio and visual modalities remains limited [20]. Unlike prior surveys, this review not only synthesizes existing methodologies but also introduces a comprehensive taxonomy that systematically organizes prior work in RD detection. In addition, two conceptual frameworks for audio-based and visual-based approaches are presented to illustrate the general pipeline commonly adopted in the literature, including data acquisition, preprocessing, feature extraction, and classification using ML and DL techniques. This structured perspective highlights research gaps, facilitates cross-modality comparison, and provides a roadmap for the development of future multimodal AI models for RD detection. In this survey, our objective is to bridge this gap by presenting an in-depth review of recent advances in RD detection across audio-based, visual-based, and audio-visual modalities. We further discuss current limitations and outline potential future research directions. To the best of our knowledge, this review is among the first to systematically examine these three domains within a single study, offering a holistic perspective on the latest developments in the field. Specifically, we propose a taxonomy of existing approaches that categorizes studies by modality (audio, visual, and audio-visual), feature extraction techniques, learning algorithms, application areas, benchmark datasets with their strengths and limitations, and evaluation metrics. This comprehensive analysis is intended to guide researchers in navigating the current landscape and identifying opportunities for innovation and improvement.

The primary contributions of this study are summarized as follows:

- We introduce a comprehensive taxonomy for detecting RD using audio-based, visual-based, and audio-visual-based modalities, leveraging ML and DL techniques.
- We provide a structured review of existing research on RD detection, covering learning approaches, application areas, benchmark datasets (along with their strengths and limitations), and evaluation metrics.
- We present audio-based and visual-based conceptual frameworks for RD detection.
- We identify gaps in the existing literature and suggest future directions for integrating audio and visual modalities to enhance the performance and efficiency of RD detection.

The remainder of this study is organized as follows: Section 2 details the review methodology for RD detection. Section 3 explores the most commonly used audio and visual modalities in the literature. Section 4 introduces a taxonomy of recent studies that utilize audio, visual, and audio-visual multi-modal approaches with AI-driven techniques. Section 5 focuses on studies employing audio-based modalities, while Section 6 examines those using visual-based modalities. Section 7 reviews recent research on hybrid audio-visual modalities for RD detection. Section 8 outlines the most commonly used evaluation metrics in the literature, and Section 9 discusses challenges and future directions. Finally, Section 10 presents the conclusions drawn from this review.

## 2. Methodology

We utilize the Preferred Reporting Items for Systematic Reviews and Meta-Analyses (PRISMA) protocol, a framework specifically developed for conducting systematic reviews [21]. The subsequent subsections detail the information sources, search terms utilized for data extraction, inclusion and exclusion criteria for determining eligibility, the process of selecting relevant articles, and the outcome obtained.

### 2.1. Data Sources

This study utilized Scopus and Web of Science (WoS) as the primary databases for searching relevant studies. These databases were selected due to their extensive coverage of peer-reviewed literature in AI, healthcare, and medical informatics, including articles published by reputable sources such as IEEE Xplore, ScienceDirect, Springer, and others. The articles from these publishers were retrieved through the indexing services of Scopus and WoS.Additionally, Google Scholar was used to supplement the search and ensure comprehensive coverage of relevant literature.

### 2.2. Data Extraction

As this review focuses on audio and visual modalities for RD detection, the literature search was divided into three categories: audio-based modalities, visual-based modalities, and audio-visual-based modalities. To ensure comprehensive coverage, consistent keywords were applied across the two databases, Scopus and Web of Science. The extracted information from the selected studies included the type of RD, dataset characteristics, modality type, preprocessing and feature extraction techniques, employed ML/DL models, and reported evaluation metrics. The extracted data were systematically organized in tabular form to facilitate structured comparison and synthesis of the reviewed studies.

Google Scholar searches were conducted using combinations of predefined keywords related to RDs, AI, and modality-specific terms. The search process employed Boolean operators such as “AND” and “OR” to improve search precision and coverage. Example keyword combinations included “respiratory diseases” AND “deep learning” AND “cough”, “respiratory diagnosis” AND “CNN” AND “X-ray”, and “multimodal respiratory disease detection” AND “AI”.

The keywords used for searching relevant studies in the Scopus and WoS databases focusing on audio-based modalities are:

“respiratory diseases” OR “respiratory conditions” OR “respiratory diagnosis”

AND

“classification” OR “detection” OR “prediction” OR “identification”

AND

“audio” OR “cough” OR “speech” OR “breath” OR “signal” OR “lung sounds”

AND

“machine learning” OR “deep learning” OR “ML” OR “DL” OR “CNN” OR “AI”.

For studies focusing on visual-based modalities, the following keywords are used to search for relevant studies in the Scopus and WoS databases.

“respiratory diseases” OR “respiratory conditions” OR “respiratory diagnosis”

AND

“classification” OR “detection” OR “prediction” OR “identification”

AND

“x-rays” OR “CXR” OR “CT scans” OR “ultrasound” OR “radiographic”

AND

“machine learning” OR “deep learning” OR “ML” OR “DL” OR “CNN” OR “AI”.

Finally, for studies using multimodal approaches that combine audio and visual modalities, the keywords used across the two databases, Scopus and WoS, are:

“respiratory diseases” OR “respiratory conditions” OR “respiratory diagnosis”

AND

“classification” OR “detection” OR “prediction” OR “identification”

AND

“multimodal” OR “multimodality” OR “audio and visual modalities”

AND

“machine learning” OR “deep learning” OR “ML” OR “DL” OR “CNN” OR “AI”.

### 2.3. Inclusion and Exclusion Criteria

The literature search was conducted between 20 March and 20 June 2025, using multiple electronic databases. The search focused on selecting articles written in English and published between 2020 and 2025 to ensure the inclusion of recent and relevant studies. This timeframe was specifically chosen due to the rapid advancement of AI and DL applications in healthcare, particularly following the COVID-19 pandemic, which accelerated research in automated RD detection using audio and visual modalities. The selection process primarily considered peer-reviewed journal articles to maintain high-quality and reliable sources. Non-relevant and preliminary conference papers were excluded to ensure the focus remains on original research and to avoid results that may lack comprehensive validation. No formal standardized risk-of-bias assessment tool was applied in this review due to the methodological diversity of AI-based studies. However, aspects such as dataset quality, validation strategies, reported evaluation metrics, and reproducibility were considered during the interpretation and comparison of the reviewed studies.

### 2.4. Studies Selection Process

**Figure 1:**
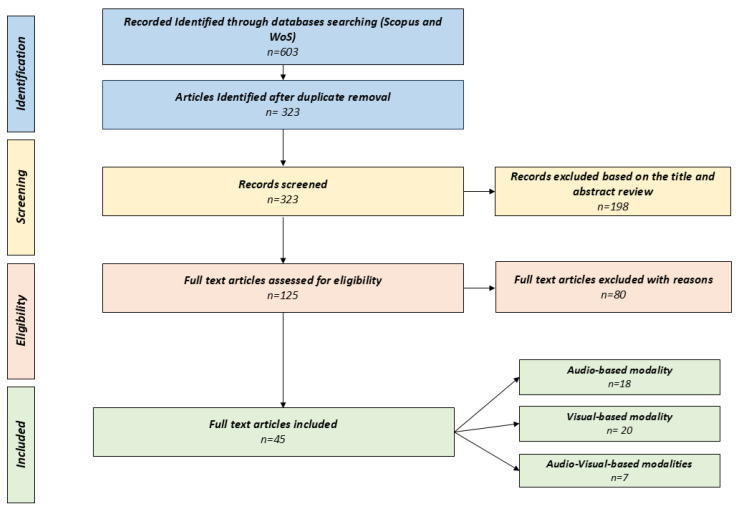
Block diagram illustrating the methodology used for selecting studies in the systematic review.

The block diagram in Figure 1 illustrates the four main steps for selecting relevant studies: identification, screening, eligibility, and inclusion of the relevant research papers. Using the outlined strategies, a total of 603 articles were initially obtained. After removing duplicates, 323 unique articles remained. The abstracts of these 323 articles were reviewed, resulting in the exclusion of 198 articles due to their lack of relevance. The remaining 125 articles underwent a thorough full-text review, which led to the exclusion of 80 articles classified as review papers, surveys, or non-relevant studies. Ultimately, 45 articles were included in the analysis and categorized into three categories: audio-based modalities (18 articles), visual-based modalities (20 articles), and audio-visual-based modalities (7 articles), taking into account modality types, learning approaches, datasets, application areas, and performance evaluation metrics. The included studies focus on ML–based models, making conventional clinical effect measures unsuitable for evaluation. Model performance was therefore assessed and summarized using commonly reported ML metrics, including accuracy, precision, recall, F1-score, specificity, and the area under the receiver operating characteristic curve (AUC). The findings were synthesized using a qualitative narrative approach supported by tabular comparison and taxonomy-based categorization. All retrieved records were screened by a single reviewer based on predefined inclusion and exclusion criteria. Studies were grouped and summarized in comparative tables to facilitate the analysis of methodological trends and performance outcomes. To assess potential risk of bias and the overall certainty of evidence, methodological aspects such as dataset quality, sample size, validation strategies, reproducibility, and consistency of reported results were examined, with studies employing rigorous validation and transparent reporting considered to provide higher confidence.

## 3. Audio and Visual Modalities in RD Detection

**Figure 2:**
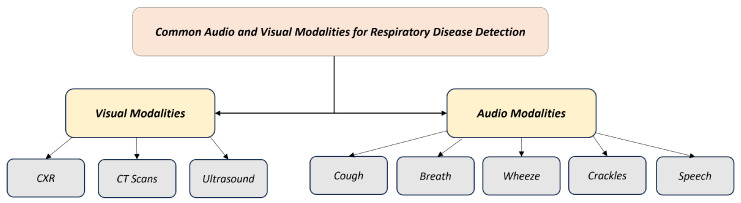
Common audio and visual modalities used for RD detection in the literature.

Figure 2 illustrates the most common audio and visual modalities explored in the literature for RD detection using AI algorithms. Respiratory sounds, such as coughs, breath sounds, crackles, wheezes, and speech, recorded through microphones or specialized stethoscopes, form the basis of audio modalities [17,22]. Audio-based approaches typically rely on acoustic features extracted from these respiratory sounds, including Mel-Frequency Cepstral Coefficients (MFCCs), spectrograms, and other time-frequency representations, which capture important spectral and temporal characteristics of the signals. These audio signals provide critical insights into respiratory system functionality by capturing anomalies such as abnormal breathing patterns or cough intensity [20]. When analyzed using ML or DL techniques, these signals enable accurate, non-invasive diagnoses of conditions like asthma, COVID-19, and pneumonia, offering a promising avenue for early detection and monitoring.

On the other hand, visual modalities, including CXR, CT scans, and ultrasound US, are widely used for detecting RDs, with CNNs commonly employed for automated feature extraction and disease classification. Each imaging modality differs in affordability, availability, and the level of expertise required for accurate interpretation [23]. For instance, CXRs and portable US units are particularly cost-effective and accessible, making them more suitable for widespread use compared to other modalities [19]. US, which utilizes high-frequency sound waves to generate real-time images of internal structures [24], is less widely available than CXRs, limiting its use in certain settings. CT scans, while capable of producing highly detailed cross-sectional images, are more expensive, less portable, and require specialized infrastructure and expertise [24].

**Figure 3:**
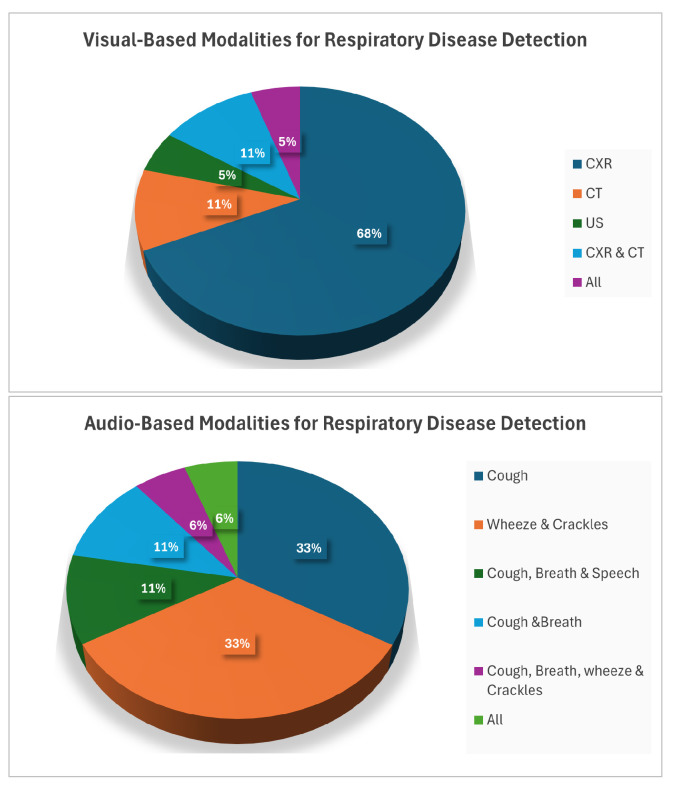
Distribution of surveyed studies by input modality for RD detection. Left: visual-based approaches; Right: audio-based approaches.

Together, audio and visual modalities offer complementary strengths, advancing the development of accessible and effective diagnostic approaches for respiratory diseases in both clinical and remote healthcare settings. The integration of these modalities with AI algorithms has the potential to significantly enhance early detection, monitoring, and treatment outcomes for respiratory conditions. Figure 3 illustrates the distribution of audio and visual modalities in the surveyed studies on respiratory disease detection. It provides insight into the most commonly employed modalities for diagnosing respiratory conditions using AI algorithms in recent years. The first chart depicts the usage rates of various visual-based modalities, including CXR, CT scans, and US images, while the second chart presents the proportions of different audio-based modalities, such as cough, breath, wheeze, and crackles, as reported in the reviewed studies.

## 4. Taxonomy of RD Detection from Audio and Visual Modalities

**Figure 4:**
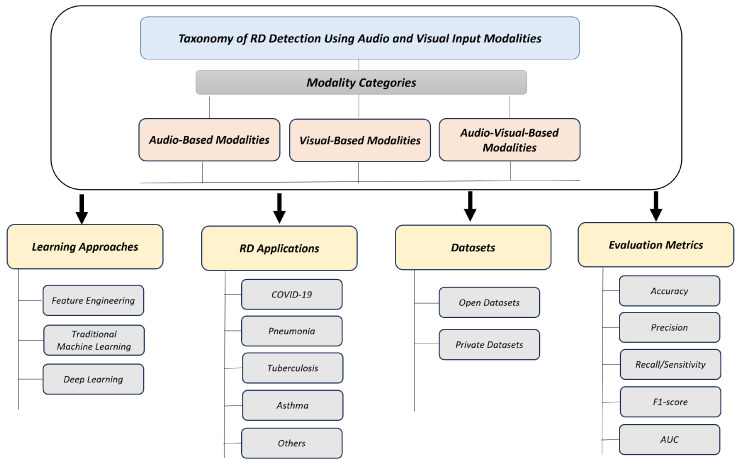
Taxonomy of RD detection using audio-based, visual-based, and audio-visual-based modalities.

This section presents a taxonomy of recent studies on RD detection that utilize audio, visual, and audio-visual modalities, leveraging ML and DL techniques. The taxonomy is designed to provide an overview and a clearer understanding of the essential concepts and key focuses of previous studies in this field, as shown in Figure 4. It categorizes the current work into three sections based on modality categories, with each section focusing on four main aspects:

- Learning Approaches: This approach is essential as it determines how meaningful features are extracted from raw data to aid in training models for the detection of RD. These training models can be based on traditional ML or DL techniques. DL models, in particular, can be utilized as-is, fine-tuned on specific datasets for targeted tasks, or developed entirely from scratch. Additionally, ensemble models, which combine predictions from multiple models, are often employed to enhance overall performance and robustness.Further elaboration on this aspect will be provided in the detailed review of each modality in the respective sections.
- RD Applications: Based on the survey literature, the most common application area is the detection of COVID-19, leveraging either audio data, such as cough sounds, or visual data, such as CXR. Similarly, tuberculosis and pneumonia are frequently predicted using visual modalities like CXR and CT scans. On the other hand, diseases such as COPD, asthma, and other respiratory conditions are often identified through audio modalities, such as wheezes and crackles, showcasing the diverse potential of these approaches in addressing various respiratory diseases. Detailed application areas are elaborated within the context of each modality in the respective sections.
- Datasets: Datasets play a crucial role in detecting RD by providing the foundation for training and evaluating models. This survey highlights the most recent and commonly used audio datasets, encompassing various modalities such as coughs, wheezes, crackles, speech, and breath sounds. Similarly, visual datasets, including CXR, CT scans and US imaging, are also discussed, emphasizing their importance in enabling accurate and robust disease detection. These datasets are presented along with their respective strengths and limitations.
- Evaluation Metrics: The effectiveness of models for respiratory disease detection is commonly assessed using metrics such as accuracy, precision, recall/sensitivity, specificity and F1-score. These metrics provide a comprehensive understanding of the model’s ability to correctly classify diseases, balance false positives and false negatives, and maintain reliability across diverse datasets. These metrics are further discussed in detail based on the surveyed literature, offering a deeper understanding of the most commonly used evaluation metrics in this area.

The literature matrix, which summarizes the key findings and methodologies of previous studies, is presented in Table 1. This provides an overview of existing work and serves as a basis for the explanation of each component in the taxonomy. The associated studies for each component will be thoroughly discussed in the subsequent sections, offering deeper insights into their contributions and applications.

## 5. Audio-Based Modalities

This section explores recent studies on RD detection using audio-based modalities, focusing on learning approaches, application areas, and recent datasets, highlighting their strengths and limitations.

### 5.1. Learning Approaches

**Figure 5:**
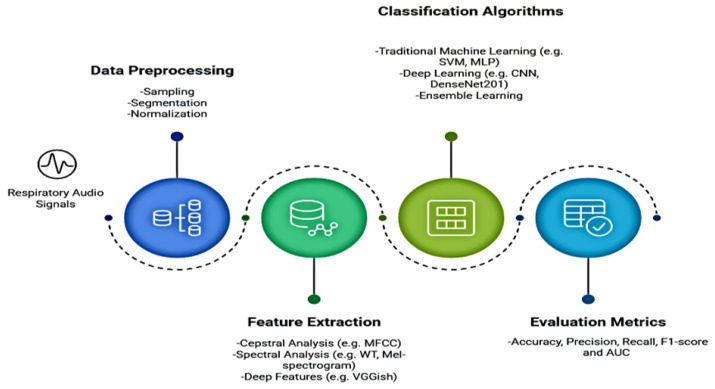
Audio-based conceptual framework for RD detection using ML and DL techniques.

A structured and well-defined learning framework needs to be followed to achieve the desired detection objectives. Figure 5 illustrates this process, which typically involves key steps such as data preparation, feature extraction, and classification, each playing a critical role in ensuring accurate and efficient detection outcomes. In respiratory audio-based analysis, various preprocessing techniques have been employed in the literature prior to feature extraction. These include filtering methods, such as Finite Impulse Response (FIR) and band-pass filters, to reduce noise [25,27,34]; sampling, with common rates ranging from 16 kHz to 48 kHz [8,22,32,33,37,44]; segmentation techniques, such as the Bayesian Information Criterion (BIC), applied within a sliding variable-size analysis window and refined using smoothing techniques [8,26,31,38]; and normalization, which enhances consistency across audio samples by adjusting amplitude levels or standardizing feature distributions [8,27,37,40].

The feature engineering phase involves transforming input signals into meaningful feature representations for model development [20]. This process enhances ML and DL performance by extracting meaningful patterns such as frequency, amplitude, and time-domain characteristics. Based on the literature, feature engineering techniques fall into two categories: handcrafted and deep features.

Handcrafted features are further divided into spectral analysis and cepstral analysis, both of which involve manually designed techniques to extract meaningful information from respiratory audio signals. Spectral analysis focuses on examining the frequency content of an audio signal and includes several widely used techniques. One such method is the Wavelet Transform (WT), which is particularly effective for analyzing non-stationary signals by decomposing them into different frequency components over time. This technique enables the extraction of both time-domain and frequency-domain information, making it well-suited for capturing subtle variations in respiratory sound patterns, such as wheezes and crackles, which are crucial for diagnosing RD [37,38]. Additionally, spectral features, such as spectral centroid, spectral bandwidth, spectral contrast, and spectral roll-off, help characterize the distribution of energy across different frequency bands, providing useful insights into respiratory sound characteristics [27,29]. Another widely adopted spectral analysis technique is the Mel-Spectrogram, which represents the spectral content of an audio signal on a perceptually meaningful scale, making it particularly useful for speech and respiratory sound analysis [22,25]. When transformed into Mel-Spectrogram images, these representations can be effectively used with convolutional neural networks (CNNs), and several studies have reported strong performance in tasks such as cough-based COVID-19 detection and respiratory sound classification [67,68]. However, the transformation and subsequent image-based processing can be computationally intensive, especially when handling large audio datasets or deploying models in real-time environments, which may limit their applicability in resource-constrained settings.

Cepstral analysis, on the other hand, examines the rate of change in spectral characteristics. The most widely used cepstral analysis technique is the Mel-Frequency Cepstral Coefficients (MFCC). Theses features are derived through a sequence of transformations, including the Fourier Transform (FT), Mel filter bank, and Discrete Cosine Transform (DCT), which together emulate the human auditory system’s perception of sound [8]. MFCCs are computationally efficient and lightweight, making them particularly suitable for real-time or embedded systems. These coefficients are frequently employed as standalone feature representations, as demonstrated in [8,34,35,36,40]. Alternatively, they can be combined with other spectral features, such as chroma, spectral centroid, spectral bandwidth, roll-off, zero-crossing rate (ZCR), and spectral contrast, to enhance analysis accuracy, as seen in studies like [27,29,32,39].

In contrast to handcrafted features, deep features leverage pre-trained models to automatically learn representations from data. This approach enables the extraction of high-level features that may not be easily captured using traditional spectral or cepstral methods. For instance, pre-trained models such as VGGish, a variant of the VGG network, have been employed for deep feature extraction, often in combination with handcrafted features, as demonstrated in [22,29]. VGG architectures, originally designed for image classification, consist of multiple convolutional layers, enabling hierarchical feature learning [69]. VGGish, an adaptation of VGG for audio processing, converts input waveforms into spectrograms and processes them through deep convolutional layers to extract discriminative feature embeddings. This hierarchical representation allows for capturing both local and global patterns in respiratory sounds, improving classification performance.

Moreover, specific studies have employed various feature selection and dimensionality reduction techniques to enhance model performance. For instance, Forward Feature Selection (FFS) was utilized in [40], while mRMR (Minimum Redundancy Maximum Relevance) was applied in [30] to identify the most relevant features while minimizing redundancy. Similarly, Principal Component Analysis (PCA) was used in [30,57,61] to reduce the dimensionality of the feature vector, improving computational efficiency and model generalization.

Prediction models in the classification stage, as identified in the reviewed studies, can be categorized into three main types. The first category includes traditional ML algorithms, such as Support Vector Machine (SVM), Decision Tree (DT), K-Nearest Neighbors (KNN), Logistic Regression (LR), Random Forest (RF), Naïve Bayes, AdaBoost, and a shallow Multilayer Perceptron (MLP) with a single hidden layer or a deep MLP with multiple layers [28,29,30,32,40]. These traditional ML models are widely used for RD classification due to their interpretability, efficiency on small datasets, and ability to handle structured feature-based inputs. However, their performance may be limited when dealing with high-dimensional data or complex patterns, where DL models often demonstrate superior accuracy and feature extraction capabilities.

The second category comprises DL algorithms, including pre-trained models such as Dense-Net201, InceptionV3, and ResNet [31,37,38], which leverage transfer learning to extract high-level features from spectrogram images of respiratory sounds. Additionally, Convolutional Neural Networks (CNNs) developed from scratch [34,49,55] are designed with customized architectures optimized for specific RD detection tasks, enabling more tailored feature extraction and classification. Additionally, Recurrent Neural Networks (RNNs) and Long Short-Term Memory (LSTM) networks have been employed for sequential data processing in respiratory sound classification [28,32]. RNNs are designed to handle sequential dependencies by maintaining internal memory, making them effective for processing respiratory sound waveforms over time. However, they suffer from issues like vanishing gradients, which can limit their ability to learn long-term dependencies. LSTMs, an advanced variant of RNNs, address this limitation by incorporating memory cells and gating mechanisms, allowing them to retain long-term information [70]. This makes LSTMs particularly useful for analyzing complex respiratory patterns and detecting anomalies in time-series audio data.

The third category is the ensemble approach, which combines multiple models to improve classification performance. This technique has been implemented in various studies [22,40] by integrating different ML or DL models to enhance robustness and predictive accuracy. Ensemble learning leverages the strengths of multiple classifiers, reducing overfitting and improving generalization. Ensemble methods often incorporate voting classifiers, including hard voting, where the final prediction is determined by the majority class label from individual models, and soft voting, where the average probability score is used to make the final decision. These techniques have been applied in RD detection, improving model stability against noisy data and enhancing overall diagnostic accuracy.

In summary, the trade-offs among different audio-based approaches highlight that no single method can be regarded as universally optimal. The suitability of a technique largely depends on the intended application: lightweight models, such as those based on MFCCs or other low-dimensional features, are preferable for mobile or point-of-care screening where efficiency and real-time operation are critical. Conversely, computationally intensive representations, such as Mel-Spectrograms images paired with deep learning architectures, may be more appropriate in centralized clinical settings where higher accuracy can justify increased resource demands.

### 5.2. Applications

RD detection has gained significant attention in recent years, driven by advancements in artificial intelligence, signal processing, and biomedical research. ML and DL techniques are increasingly being used to analyze respiratory sounds, facilitating the early diagnosis and monitoring of conditions such as COVID-19, asthma, chronic obstructive pulmonary disease (COPD), and pneumonia. The reviewed studies below provide a deeper insight into these applications.

COVID-19: Grounded in existing studies, COVID-19 detection has emerged as a leading application of ML in RD analysis, with numerous studies exploring the use of cough, breath, and speech sounds for early diagnosis and monitoring. Several studies [8,22,25,27,30,32,33] have explored the detection of COVID-19 using cough sounds, as coughing is a key biomarker of the disease, along with breathing and speech using open-source datasets [71,72,73,74,75]. These studies demonstrate that ML models can serve as pre-screening tools, enabling rapid and cost-effective identification of potential cases. Such models assist healthcare professionals in prioritizing patients for further testing, reducing the strain on healthcare systems, and improving early intervention efforts.

Asthma: Another area of RD applications where AI algorithms have shown great promise is asthma detection and monitoring. Aptekarev et al. [31] proposed a computer-assisted diagnostic model for asthma based on respiratory sound analysis. Using data from a Russian children’s hospital, the model classifies sounds from various anatomical points to differentiate asthma stages (exacerbation, well-controlled, partially controlled, and uncontrolled). This approach enhances early detection, continuous monitoring, and accessible asthma care in both clinical and home settings. Moreover, Abadade et al. [34] developed a TinyML-based model for asthma diagnosis, using an optimized CNN to distinguish asthma from non-asthma conditions via lung sound recordings. Designed for deployment on low-power, cost-effective devices, this approach enhances real-time, portable asthma diagnosis, making it accessible in resource-limited settings.

COPD: COPD presents significant challenges when diagnosed at a late stage, potentially leading to severe deterioration in a patient’s quality of life and increasing the strain on healthcare systems. Two studies [38,40] explored COPD detection as a key application area, utilizing wheeze and crackle sound analysis to differentiate between COPD-affected lungs and healthy individuals. The proposed models demonstrate substantial improvements in identifying COPD patients and assessing respiratory illness risks, highlighting the effectiveness of AI-driven approaches in early diagnosis and disease management.

Pneumonia: Pneumonia detection is another area of research that leverages cough sound analysis as an input modality. Two studies [26,29] have proposed AI-driven solutions to distinguish childhood pneumonia from bronchitis, using an open-source dataset [26] collected from 173 patients at West China Second University Hospital. These studies employ ML techniques to analyze respiratory sounds, extracting meaningful features for accurate classification. Their findings underscore the potential of AI in early pneumonia detection, improving diagnosis and patient management.

Lung Diseases: The classification of multiple lung diseases has been widely explored in the literature, with several studies focusing on distinguishing five common lung disorders using open-source datasets containing wheeze and crackles sound recordings [28,35,36,37,39]. Research efforts have particularly addressed the classification of asthma, pneumonia, Lower Respiratory Tract Infection (LRTI), Upper Respiratory Tract Infection (URTI), and COPD using open-source datasets [76,77]. The solutions proposed in these studies highlight the potential of DL and ML in medical diagnostics for lung disorders through respiratory sound analysis of wheezes and crackles, paving the way for advancements in AI-driven precision medicine and the development of intelligent healthcare systems.

### 5.3. Audio-Based Datasets

Given the growing demand for audio-based datasets, several open-source datasets have been introduced, particularly during the COVID-19 pandemic, providing valuable resources for research and development. A brief overview of these datasets is provided below, while Table 2 highlights their key strengths and limitations.

-Respiratory Sound Database (ICBHI) (2019): The database, published in 2019, contains 920 recordings from 126 participants [76]. A total of 6898 respiratory cycles were captured and annotated by three experienced physicians, with recording durations varying between 10 and 90 s. This dataset, utilized in studies [35,36,37,39,40], encompasses both chronic and non-chronic respiratory conditions along with healthy control samples.

-Coswara Dataset (2020): The dataset was collected through crowdsourcing and comprises approximately 1,000 respiratory sound samples, including coughs, breaths, and voice recordings [73]. It also includes metadata such as age, gender, location, and health status. This dataset, which has been used in studies [30,33], is designed to support and enhance PCR-based COVID-19 diagnostic methods.

-Virufy Dataset (2020): The dataset was collected under medical supervision in a hospital setting [71] and has been utilized in [8] to differentiate COVID-19-infected patients from healthy individuals. It includes 121 segmented cough samples from 16 patients, labeled with PCR-confirmed COVID-19 status and patient demographics. Data are organized into original and segmented recordings to facilitate efficient processing and classification.

-The Covid19-Cough Dataset (2020): The dataset consists of 1,324 samples, including 682 COVID-19 positive cases [74], and has been used in several studies [22,33] for COVID-19 detection from respiratory recordings. The recordings are classified into symptomatic COVID-19 positive, asymptomatic COVID-19 positive, and COVID-19 negative cases. Data collection was carried out via a call center and a Telegram bot, ensuring broad accessibility and diverse participant contributions.

-NeurIPS Dataset (2020): The dataset, collected by a team from the University of Cambridge [67], consists of crowdsourced cough and breathing recordings aimed at facilitating automatic COVID-19 diagnosis. It has been used in [27] to distinguish COVID-19 patients from those with asthma and healthy individuals. Data was collected via a web-based platform and an Android app, enabling participants to submit recordings along with symptom reports and COVID-19 test results. This resulted in 4352 unique users from the web app and 2261 unique users from the Android app. Among them, 235 participants reported testing positive for COVID-19 (64 via the web app and 171 via the Android app).

-COUGHVID Dataset (2021): The dataset comprises over 25,000 cough recordings from a diverse group of participants, accompanied by rich metadata, including gender, age, and medical history [72]. It is categorized into three groups: healthy, asymptomatic, and COVID-19 positive, and has been utilized in several studies [8,22,33] for COVID-19 classification tasks. A portion of the dataset was analyzed by expert physicians to assess cough severity and identify medical abnormalities, such as respiratory infections and obstructive diseases.

-King Abdullah University Hospital Dataset (KAUH) (2021): The dataset consists of 310 recordings from 105 patients diagnosed with various respiratory conditions, including asthma, pneumonia, heart failure, bronchiectasis, and COPD [77]. Recordings were collected using a 3M Littmann Electronic Stethoscope Model 3200, placed at different chest wall locations, with detailed annotations specifying sound types such as inspiratory, expiratory, wheezes, crackles, and normal breath sounds. This dataset has been utilized in [34] to classify various lung diseases.

-The Second DiCOVA Challenge Dataset (2021): The dataset comprises audio recordings of breathing, cough, and speech from COVID-19 positive and negative individuals [75] and has been utilized in [27] to detect COVID-19 cases using multiple audio modalities. The development set includes data from 965 participants (172 COVID-19 positive), while the evaluation set consists of 471 participants (71 COVID-19 positive).

-The Sarcos Dataset (2021): The SARCOS dataset was collected in South Africa as part a research project [78]. Participants voluntarily recorded their coughs using an online platform after undergoing a SARS-CoV laboratory test. The dataset includes cough audio samples from 18 COVID-19 positive and 26 COVID-19 negative individuals, recorded at a 44.1 kHz sampling rate. This dataset has been used in [62] to automate COVID-19 detection from audio recordings, alongside a visual CXR dataset.

-The SPRSound Dataset (2022): The SPRSound dataset is the first open-access pediatric respiratory sound database, comprising 2683 recordings from 292 participants aged 1 month to 18 years [79]. It includes respiratory conditions such as asthma, bronchitis, pneumonia, and other respiratory diseases. Data were collected at Shanghai Children’s Medical Center using stethoscopes and annotations were meticulously performed by 11 experienced pediatric physicians, ensuring high-quality labeling. It has been used in [36] to enhance lung acoustic signal classification.

-West China Second University Hospital Dataset (2022): The dataset consists of cough recordings from 173 children (ages from 0 years to 11 years) at the West China Second University Hospital, China, categorized into pneumonia and non-pneumonia cases [26]. The pneumonia class includes bronchopneumonia and lobar pneumonia, while the non-pneumonia class comprises bronchitis and bronchiolitis. The recordings were captured as MP3 files in a pediatric consultation room, with the recorder positioned 20 to 40 cm from the patient’s mouth. The average audio duration per patient is 3.92 s, and background noise may be present due to accompanying family members. This dataset has been utilized in studies [26,29] for the automated detection of childhood bronchitis and pneumonia.

## 6. Visual-Based Modalities

This section reviews recent research on RD detection using visual-based modalities, focusing on learning approaches, practical applications, and the latest datasets utilized in the literature, along with their strengths and limitations.

### 6.1. Learning Approaches

**Figure 6:**
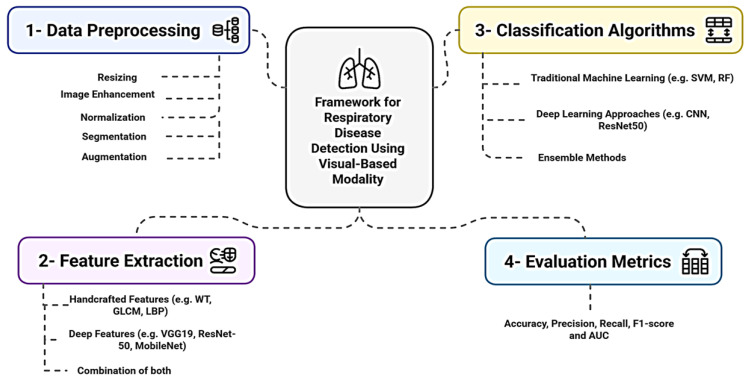
Visual-based conceptual framework for RD detection using ML and DL techniques. The dashed line indicates a conceptual relationship between the corresponding framework components.

Similar to audio-based modalities, visual-based modalities require a structured and well-defined learning framework to achieve accurate detection. As illustrated in Figure 6, this process typically consists of key stages, including data preparation, feature engineering, and classification, which are then evaluated using evaluation metrics.

Preprocessing techniques play a crucial role in enhancing the quality of visual data for respiratory disease detection. These techniques include resizing to standardize image dimensions, normalization to scale pixel values, image enhancement to improve visual clarity, lung segmentation to isolate relevant regions, and augmentation to increase dataset diversity and improve model robustness. Several studies have applied resizing to ensure dataset consistency and enhance model performance [44,47,48,49]. Additionally, few studies have utilized normalization to scale pixel values within a specific range, which helps stabilize model training and improve convergence [46,49].

Data enhancement is another preprocessing technique used in the literature to improve dataset quality and enhance model performance. To enhance lesion visibility, Bhimavarapu et al. [51] applied an Enhanced Gaussian distribution to improve the visual clarity of affected regions, making abnormalities more distinguishable. Some studies have implemented Contrast-Limited Adaptive Histogram Equalization (CLAHE) to enhance image contrast while preserving pixel intensity consistency, particularly benefiting medical imaging applications [23,45]. Segmentation techniques have been utilized to precisely identify diseased regions, enhancing detection accuracy. U-Net, a widely used approach, employs an encoder-decoder architecture with skip connections to preserve spatial information while capturing high-level features, improving performance by focusing on the most relevant areas of interest. As shown in studies [42,50,55], segmentation methods aid in isolating affected areas, minimizing noise, and optimizing the efficiency of subsequent classification processes. However, these techniques can be computationally expensive and require high-quality annotations. Moreover, few studies have utilized common augmentation techniques, such as flipping, rotation, shearing, and shifting, which create variations of existing images and help models become more robust against overfitting [46,56,57]. Furthermore, Dasanayaka and Dissanayake in study [42] applied image augmentation using Deep Convolutional Generative Adversarial Networks (DC-GANs) to synthetically generate new training samples, further increasing dataset variability and enhancing model performance.

The feature engineering phase is a critical step in visual modalities, playing a key role in extracting meaningful patterns to be used in the subsequent classification stage for RD detection. According to previous studies, feature extraction techniques can be broadly classified into handcrafted features and deep learning-based features. Several studies [45,50,59] have employed handcrafted feature extraction methods, such as WT for image decomposition. Additionally, statistical feature extraction methods have been utilized, including the Gray-Level Co-occurrence Matrix (GLCM), Gray-Level Difference Matrix (GLDM), Neighborhood Gray-Tone Difference Matrix (NGTDM), Gray-Level Run Length Matrix (GLRLM), Gray-Level Size Zone Matrix (GLSZM), and Local Binary Patterns (LBP). These features are useful for capturing texture and spatial relationships within medical images, thereby enhancing classification performance. They can be used as standalone features with traditional ML classification algorithms such as SVM, RF, LR, DT, KNN, and Gradient Boosting (GB) [45,50]. Feature selection techniques, including PCA, or Mutual Information (MI), are often applied to optimize the selection of relevant features. Besides, these handcrafted features can be combined with deep learning-based features, as shown in [55,59], to improve classification performance. Other studies have utilized ensemble approaches for feature extraction and classification, as seen in [42,58,59], which integrate multiple models to enhance predictive accuracy and robustness.

On the other hand, deep learning-based feature extraction and classification are widely used in the literature to derive abstract and high-level features, such as edge patterns, textures, shapes, spatial hierarchies, and semantic representations [23,41,44,46,48,49,51,52,54,55,57,62,66]. These techniques leverage convolutional layers to automatically learn hierarchical representations, where lower layers detect simple features (e.g., edges and textures), and deeper layers capture more complex structures (e.g., shapes and spatial relationships). For instance, several studies [23,52,62,66] have employed pretrained deep learning models such as VGG19, ResNet50, InceptionV3, Xception, and MobileNet for feature extraction and classification. These models, trained on large-scale datasets, transfer learned feature representations to RD detection, enhancing classification accuracy while reducing training time. Meanwhile, other studies [41,48,51,54] have developed custom CNN architectures from scratch to learn task-specific features directly from medical images, allowing for greater adaptability to unique dataset characteristics but requiring more extensive training and hyperparameter tuning.

### 6.2. Applications

Based on our survey, recent studies on the application of visual-based modalities in detecting RD have primarily focused on three main areas over the last five years: COVID-19, tuberculosis (TB), and pneumonia. The following reviewed studies provide a thorough investigation of these applications.

- COVID-19: The detection of COVID-19 has become a significant application of ML in RD analysis over the past few years. Numerous studies have explored the use of CXR, CT scans, and US images for early diagnosis and patient monitoring. Among these, the majority of studies [23,41,44,46,52] have proposed various methods for detecting COVID-19 using CXR, which is widely accessible, cost-effective, and efficient—especially in resource-constrained environments where advanced imaging techniques like CT scans may not be readily available. Ragab et al. [46] introduced a novel Quantum Seagull Optimization Algorithm with a Deep Learning-based COVID-19 diagnosis model (QSGOA-DL) to detect and classify COVID-19 from CXR images. Their findings suggest that the proposed QSGOA-DL technique can serve as a reliable tool for COVID-19 diagnosis using CXR images. Similarly, Abbasi et al. [44] developed COVIDX, a machine learning-based model designed to diagnose COVID-19 infection and assess its severity from digital CXR images. Their results indicate that COVIDX is effective in both detecting COVID-19 and predicting its severity, making it a practical tool for real-world healthcare applications. Few studies [45,55] have proposed diagnostic solutions using the open-source SARS-CoV-2 CT-Scan Dataset [80]. These approaches, primarily based on statistical analysis such as WT, GLCM, and LBP, suggest that the proposed methods are effective for COVID-19 detection. Furthermore, they suggested that these methods could be extended to diagnose various infections using CXR, and other medical imaging modalities. Additionally, Awasthi et al. [43] introduced Mini-COVIDNet, an efficient and lightweight deep neural network designed for ultrasound-based point-of-care COVID-19 detection using the open-source dataset POCUS [81]. This model emphasizes smaller networks capable of running on mobile or embedded systems, enabling immediate detection without the need for additional computing hardware.
- Pneumonia: The use of visual-based modalities, such as CXR and CT scans, plays a crucial role in pneumonia detection research. Several studies [49,51,53,56,59] have proposed automated pneumonia diagnostic solutions to assist in the absence of a radiologist. Specifically, studies [49,53,56] focus on developing pediatric pneumonia diagnosis models using CXR images from the open-source Kermany dataset [82]. Trivedi and Gupta [49] introduced a lightweight deep learning architecture for automatic pneumonia detection, leveraging the pre-trained MobileNet model. Gowri et al. [56] employed a Masked Neural Network (MNN) and KNN for pneumonia detection, while Arya and Kumar [53] utilized a CNN-based approach for the same task. Meanwhile, Shati et al. [59] proposed a comprehensive fusion model combining KNN, WT, GLCM, and ResNet50, designed to predict pneumonia in both adult and pediatric patients. Their findings indicate that these AI-driven solutions can reliably diagnose pneumonia and may be particularly beneficial in rural areas or regions with limited access to radiological expertise. Additionally, Bhimavarapu et al. [51] developed an automated system to assess pneumonia severity using both CXR and CT scan images, further enhancing the potential for AI-based pneumonia diagnosis and management.
- Tuberculosis: TB detection through visual- based modalities using AI algorithms has been an active and promising research area in recent years. Many studies [42,47,48,54,58] have developed DL solutions using commonly accessible open-source TB CXR image datasets [83,84]. In contrast, Hag et al. [50] proposed a machine vision-based approach for detecting lung tuberculosis using CT scan images. Their study utilized a private dataset comprising 100 abnormal (TB-infected) and 100 normal CT scan images of lungs, acquired from Bahawal Victoria Hospital (BVH) Bahawalpur, Department of Radiology. The scarcity of studies leveraging CT scans for TB detection is notable due to the limited availability of CT scan image datasets. Nevertheless, the findings of these studies are promising, demonstrating good performance across different datasets and highlighting the potential for practical applications in computer-aided diagnosis systems for TB detection from CXR and CT scan images.

### 6.3. Visual-Based Datasets

**Figure 7:**
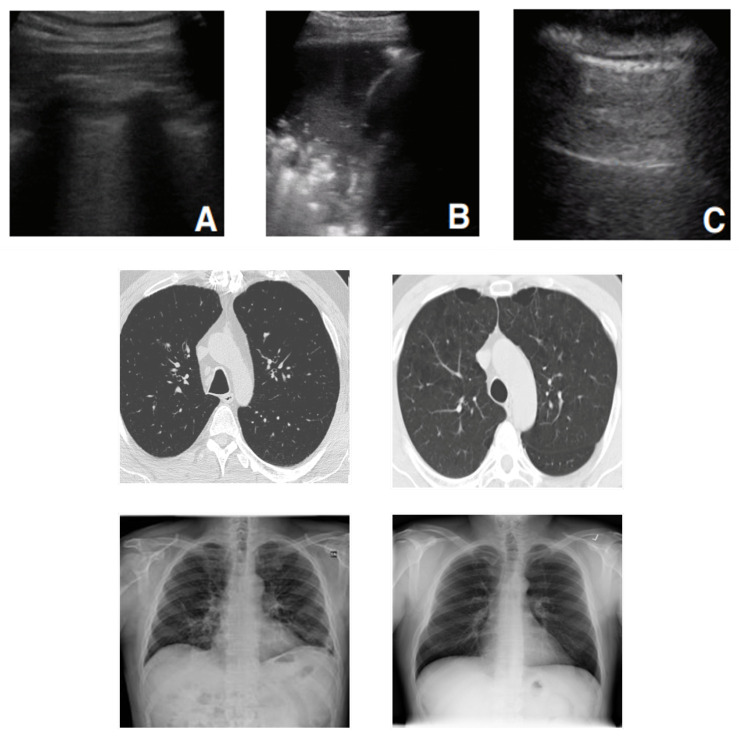
Visual-based modality types include: First row—Ultrasound (US) images representing (A) COVID-19-infected lung, (B) pneumonia, and (C) normal lung, respectively [81]. Second row—CT scan images of COVID-19 (left) and Non-COVID-19 (right) [85]. Third row—Chest X-ray (CXR) images of COVID-19 (left) and normal (right) [86].

This section highlights the most commonly used open-source datasets in the literature for visual-based modalities, including CXR, CT scans, and US imaging. Figure 7 provides examples of these three visual modalities, adapted from datasets [81,85,86]. A brief overview of open-source visual-based modality datasets is provided below, while Table 3 and Table 4 present key details, including modality types, key advantages, and limitations.

- Shenzhen Dataset (2014): The dataset was collected in collaboration with Shenzhen No.3 People’s Hospital and Guangdong Medical College in Shenzhen, China [83]. The CXR images were collected from outpatient clinics as part of routine hospital procedures. The dataset includes 662 frontal CXR images, comprising 326 normal cases and 336 with TB manifestations, including pediatric anteroposterior (AP) images. It has been used in [42,47,48] to automate pulmonary TB detection from CXRs.
- Montgomery County Chest X-ray Set (MC) (2014): The MC dataset was compiled in partnership with the Department of Health and Human Services in Montgomery County, Maryland, USA [83]. This dataset, used in studies [42,47,48], consists of 138 frontal chest X-rays from the county’s tuberculosis screening program, including 80 normal cases and 58 with TB manifestations.
- NIH Chest X-ray (ChestX-ray8) Dataset (2017): This hospital-scale CXR database contains 108,948 frontal-view X-ray images from 32,717 unique patients [16]. Each image is annotated with text-mined labels for eight different diseases, enabling multi-label classification, along with normal cases. The labeled diseases include Atelectasis, Cardiomegaly, Effusion, Infiltration, Mass, Nodule, Pneumonia, and Pneumothorax. The dataset has been used in studies [23,41] to detect various lung diseases.
- Kermany Dataset (2018): The dataset includes 5856 posterior-to-anterior (PA) CXR images from pediatric patients aged one to five years [82]. Kermany et al. collected these images at the Guangzhou Women and Children’s Medical Center as part of routine clinical care. The CXR images are labeled as pneumonia (bacterial or viral) or normal cases. This dataset has been used in studies [49,53] to automate pediatric pneumonia detection from CXR.
- RSNA Pneumonia Detection Challenge (2018): The dataset contains 30,227 CXR images in DICOM format [87]. It was sourced from the National Institutes of Health (NIH) and has been labeled by the Radiological Society of North America (RSNA) in collaboration with the Society for Thoracic Radiology and MD.ai. The images represent adult patients diagnosed with either Pneumonia (20,672 cases) or Non-Pneumonia conditions (9555 cases). This dataset has been used in studies [59,63] to develop AI algorithms for automated pneumonia detection from CXR images.
- PadChest Dataset (2020): This dataset comprises over 160,000 CXR images from 67,000 patients, collected and analyzed by radiologists at Hospital San Juan (Spain) [88]. The associated reports include 174 radiographic findings, 19 differential diagnoses, and 104 anatomical locations. A portion of the reports was manually annotated by trained physicians, while the rest were labeled using a supervised approach incorporating a recurrent neural network (RNN). It has been used in [63] alongside an audio dataset to detect COVID-19 from both audio and visual modalities.
- Tuberculosis (TB) Chest X-ray Database (2020): This dataset, developed by researchers from Qatar University and the University of Dhaka, in collaboration with Hamad Medical Corporation and partners from Malaysia and Bangladesh, contains CXR images for TB detection [84]. Sourced from multiple datasets, it includes 700 publicly available TB images, 2800 TB images accessible through the NIAID TB portal with an agreement, and 3500 normal images. It has been used in [54,58] to classify TB-infected and normal cases from CXR images.
  -COVIDx (2020): This open-access benchmark dataset comprises 84,818 CXR images from 45,342 subjects [89]. It has been used in [44] for COVID-19 prediction and severity assessment. The dataset includes separate validation and test sets, containing both COVID-positive and COVID-negative cases. Continuously updated from 2020 to 2023, it has incorporated additional CXR images over time.
- COVID-19 image data collection (2020): The dataset includes CT scans and CXR images, aimed at detecting COVID-19 [90]. It comprises 761 images from 412 individuals across 26 countries, with 679 frontal and 82 lateral CXR views.

This dataset was created by aggregating medical images from public sources, including websites and publications, to support the development and evaluation of ML models for COVID-19 diagnosis. It represents a globally diverse dataset of COVID-19 cases and has been used in studies such as [23,44] for COVID-19 detection using CXR and CT scans.

- POCUS (2020): The dataset includes 64 lung ultrasound (US) videos: 11 from healthy patients, 14 from pneumonia cases, and 39 from COVID-19 patients [81]. Collected from various sources, the videos exhibit variations in format and illumination. A total of 1103 lung US images were extracted, consisting of 182 healthy, 277 pneumonia, and 678 COVID-19 images. The dataset has been used in studies such as [23,43] to develop efficient ML models for COVID-19 detection.
- SARS-CoV-2 CT-Scan Dataset (2020): This publicly available CT scan dataset was collected from real patients in hospitals across São Paulo, Brazil [80]. It contains 1252 scans from COVID-19 positive patients and 1230 scans from non-infected individuals, totaling 2482 scans. The dataset has been used in studies [45,52,55] to automate the detection of COVID-19 from CT scans.
- COVID-19 CT Scans (2020): The COVID-CT-Dataset consists of 349 CT images with clinical findings of COVID-19, collected from COVID-19-related papers published in medRxiv, bioRxiv, NEJM, JAMA, and other sources [85]. The dataset, used in [23], includes images from 216 patients, comprising both COVID-19 positive and non-COVID cases, along with patient meta-information.
- COVID-19 Radiography Database (2021): This CXR image database includes COVID-19 positive cases, as well as normal and viral pneumonia cases [86]. It has been used in [52] to differentiate COVID-19 from pneumonia and normal cases. The dataset was compiled from multiple sources through a collaborative effort by researchers from Qatar University (Doha, Qatar) and the University of Dhaka (Bangladesh), in partnership with institutions from Pakistan and Malaysia, and in consultation with medical professionals. It comprises 3616 COVID-19 positive cases, 10,192 normal cases, 6012 lung opacity cases (representing non-COVID lung infections), and 1345 viral pneumonia cases, along with their corresponding lung masks, facilitating segmentation tasks and enhance model interpretability.
- Lung X-Ray Image Dataset (2023): The dataset, sourced from various hospitals, clinics, and healthcare institutions, comprises 3475 CXR images [91]. It is categorized into three classes: Normal (1250 images of healthy lungs), Lung Opacity (1125 images depicting lung abnormalities), and Viral Pneumonia (1100 images of viral pneumonia cases). It has been used in [57] to automate the diagnosis of various lung diseases.

**Table 3:** Strengths and Limitations of Open-Source Visual-Based Datasets for RD Detection (Part 1).

Dataset	Modality Types	Strengths	Limitations
Shenzhen [83]	CXR	Focused dataset for pulmonary TB detection enabling benchmarking.	Limited sample size (662 CXR). Image quality variability may affect consistency.
MC [83]	CXR	High-resolution images support detailed lung analysis. Lung segmentation masks aid AI-based studies.	Small sample size (138 CXR). Single-source U.S. data limits diversity.
ChestX-ray8 [16]	CXR	Large, diverse dataset covering eight RD. Real-world hospital data.	Weak labels from automated text extraction.
Kermany [82]	CXR	Pediatric pneumonia-focused dataset.	Single-center collection. Missing patient metadata.
RSNA [87]	CXR	Radiologist-annotated pneumonia dataset.	Annotation variability across experts.
PadChest [88]	CXR	Multi-label thoracic disease dataset.	Mixed manual and automated labels.
COVID-19 Image Data [90]	CXR, CT	Globally diverse dataset with detailed annotations.	Multi-source aggregation may introduce bias.

**Table 4:** Strengths and Limitations of Open-Source Visual-Based Datasets for RD Detection (Part 2).

Dataset	Modality Types	Strengths	Limitations
POCUS [81]	US	Rare ultrasound dataset for RD detection.	Small dataset size. Source variability affects performance.
(TB) Chest X-ray [84]	CXR	TB-specific dataset supporting reproducibility.	Multi-source image variability. Missing patient metadata.
COVIDx [89]	CXR	Multinational data improves generalizability.	Inconsistent image quality. Limited metadata.
SARS-CoV-2 CT-Scan [80]	CT	Clinically relevant patient scans.	Single-center dataset. Limited metadata.
COVID-19 CT Scans [85]	CT	Radiologist-validated annotations. Includes patient metadata.	Multi-source image inconsistency. Small dataset size.
COVID-19 Radiography [86]	CXR	Differentiates COVID-19, pneumonia, and normal cases.	Variable image quality. Missing metadata.
Lung X-Ray Image [91]	CXR	Diverse cases for RD detection.	No patient metadata.

## 7. Audio-Visual-Based Modalities

Audio-visual-based modalities provide a comprehensive approach to RD detection by integrating audio signals (such as cough, speech, breath sounds, wheezes, and crackles) with visual data (including CXR, CT scans, and US images). By combining information from multiple sources, this approach improves diagnostic performance and facilitates early disease detection. Compared with unimodal systems, multimodal approaches have the potential to capture a broader range of physiological and pathological characteristics, thereby improving diagnostic reliability and reducing modality-specific limitations. However, multimodal integration remains relatively underexplored in literature due to several challenges, including the scarcity of synchronized paired datasets, variability in data acquisition protocols, increased computational complexity, and difficulties associated with multimodal feature alignment and fusion. This section reviews recent studies on audio-visual-based methods for RD detection, highlighting learning approaches, applications, and the latest datasets used in the literature.

### 7.1. Learning Approaches

Integrating audio and visual modalities for RD detection became a growing research focus during the early stages of the COVID-19 pandemic. It follows a similar framework as individual audio- or visual-based approaches, involving preprocessing, feature extraction, and classification, but combines both modalities through feature fusion or decision fusion to enhance predictive accuracy.

Preprocessing techniques include sampling, segmentation, and filtering of medical images and respiratory sounds [60,64,65]. These steps are crucial for enhancing data quality, reducing noise, and improving feature extraction for classification tasks. One such technique used in the literature is the U-shaped Dual Swin Attention Transformer (UDST), a deep learning-based model designed for CXR segmentation, which leverages attention mechanisms to enhance spatial feature representation and segmentation accuracy [65]. Furthermore, filtering techniques like Anisotropic Diffusion and the Fast Guided Filter have been employed to minimize noise while preserving critical edges, ensuring that fine anatomical structures remain intact during decomposition [65]. For respiratory sound analysis, noise reduction methods have been applied to enhance audio quality by suppressing background interference, improving the clarity of pathological respiratory signals [64].

Feature engineering is widely utilized in research [60,61,62,63,64,65,66] to extract meaningful patterns from both audio and visual modalities. Studies employ different approaches for feature extraction, primarily categorized into two methods. The first approach involves converting audio signals, such as coughs and breathing sounds, into spectrograms or mel-spectrograms, allowing them to be treated as images. Deep learning models such as ResNet50, VGGsh, Inception are then applied to extract features directly from both converted audio representations and CXR images, enhancing diagnostic performance [61,63,66]. The second approach relies on handcrafted feature extraction, where MFCCs are commonly used to analyze respiratory audio signals [60,62,64,65]. For visual modality analysis, some studies employ deep learning-based feature extraction methods, such as CNN from scratch, U-Net, and Darknet, which utilize convolutional layers to automatically learn spatial hierarchies of features, enhancing disease classification [60,62]. Others rely on handcrafted visual features, including Texture Gaussian space features, which model intensity variations and spatial structures, and histogram features, which capture pixel intensity distributions to distinguish pathological patterns [64]. Additionally, advanced techniques like Deep Graph Regularized Nonnegative Matrix Factorization (DGNMF) have been employed to analyze segmented images by factorizing image data into meaningful components while preserving intrinsic geometric structures, effectively capturing intricate relationships within the data [65]. These diverse approaches highlight the ongoing advancements in feature engineering, aiming to improve the accuracy and robustness of RD detection through multimodal learning.

In the classification stage, the approach differs based on how predictions are generated. Some studies [60,62,64,66] utilize ensemble models, where predictions from separately trained models on audio and visual modalities are combined to improve accuracy. Various techniques are employed for prediction integration, including the weighted sum rule fusion-based method [62] and ML-based fusion models, which aggregate outputs from independently trained models to generate the final prediction, following the late fusion strategy. Alternatively, other studies adopt a single model trained on fused features from both modalities, incorporating the early fusion strategy, allowing the model to learn joint representations of audio data (e.g., cough sounds, breath sounds) and visual data (e.g., CXR, CT scans) [61,63,65]. This early fusion approach enables the model to capture intricate relationships between acoustic and imaging features, potentially leading to more comprehensive and robust RD detection. However, the choice between late fusion and early fusion strategies often depends on computational complexity and the specific diagnostic objectives of the study.

### 7.2. Applications

According to our survey, this approach is still developing, with most studies focusing on COVID-19 detection, while only one study has explored its application for COPD detection. These two application areas, along with their associated studies, are elaborated on below.

- COVID-19: COVID-19 remains a compelling research focus, with recent advances that incorporate both audio and visual data for enhanced analysis. Several studies [60,61,62,63,65,66] have proposed various solutions for COVID-19 detection using audio signals (such as cough and breath sounds) and visual modalities (such as CXR and CT scans). Studies [60,61,62,65] have utilized open-source audio datasets, including Coswara, Virufy, and COUGHVID [71,72,73], as well as visual datasets, such as the SARS-CoV-2 CT-scan dataset and the COVID-19 Radiography Database [80,86]. Meanwhile, other studies [63,66] have collected private audio datasets in addition to leveraging open-source audio and visual datasets. The proposed solutions highlight the novel methodologies explored in these studies and their potential impact on enhancing early diagnosis and management of COVID-19. By leveraging optimized networks and advanced techniques, these approaches offer effective solutions for COVID-19 detection from multiple inputs, ensuring a reliable and accurate diagnosis.
- COPD: In a 2024 study, Kumar et al. [64] examined the application of audio-visual-based modalities for COPD detection. Their study proposed a novel multimodal framework for the early diagnosis and classification of COPD, integrating CT scan images and multivariate pulmonary respiratory signals. The dataset was privately collected from AIIMS, Raipur, Chhattisgarh. Their approach demonstrated high performance by employing a weighted sum-rule fusion method, which integrates predictions from separately trained models. The study highlights the lack of prior research on multimodal DL frameworks for early COPD diagnosis. However, the authors believe their proposed approach can facilitate a fast, automated diagnostic system for assessing COPD severity using cough and lung sound signals in clinical settings, ultimately benefiting healthcare and society.

### 7.3. Audio and Visual Based Datasets

There is a notable lack of datasets that include both respiratory audio and visual modalities from the same patients. While researchers have leveraged open-source datasets to advance this field, truly comprehensive multimodal datasets remain scarce. The datasets explored in this survey, which have been utilized in the literature for visual and audio modalities, are discussed in Section 5.3 and Section 6.3, including [71,72,73,78,80,86,87,88,90]. These resources contribute to AI-driven diagnostic advancements, yet the development of more extensive multimodal datasets is essential for further progress in this domain.

## 8. Evaluation Metrics

Based on our survey of the literature, almost all studies have employed supervised learning algorithms to predict RD through audio and visual modalities, either as binary classification (e.g., diseased vs. healthy) or multi-class classification (e.g., different respiratory conditions), depending on the available data labels. The most commonly used evaluation metrics in the literature include accuracy (Acc), F1-score, precision, recall (sensitivity), and, in some cases, specificity. Table 5 provides a summary of these metrics, showing the aspects of model performance they capture. Accuracy is a fundamental metric that has been used in many studies to measure the proportion of correctly classified instances among all samples [8,25,28,30,31,34,35,37,38,39,40,42,45,46,47,48,50,51,52,53,55,56,58,60,61,62,63,64]. It is calculated as:

(1) $$Accuracy=\frac{TP+TN}{TP+TN+FP+FN}$$

where TP (True Positives), TN (True Negatives), FP (False Positives), and FN (False Negatives) represent classification outcomes. However, while Acc provides an overall measure of performance, it is not always sufficient—especially when dealing with imbalanced datasets where one class dominates.

To address this limitation, additional metrics such as precision and recall (sensitivity) are crucial. For example, the precision metric has been reported in several studies [8,34,35,37,40,43,48,49,53,54,56,58,60,64] to measure the proportion of correctly predicted positive cases out of all predicted positive cases. High precision indicates a low false positive rate, which is essential in preventing unnecessary medical interventions. It is defined as:

(2) $$Precision=\frac{TP}{TP+FP}$$

On the other hand, recall (sensitivity), which has been reported in studies [8,34,35,37,40,42,43,45,46,47,48,49,53,54,55,56,58,60,64], measures how well the model identifies actual positive cases. It is defined as:

(3) $$Recall=\frac{TP}{TP+FN}$$

The F1-score is the harmonic mean of precision and recall, balancing both metrics and providing a more reliable assessment, particularly when dealing with imbalanced datasets. Several studies [28,33,34,38,40,43,44,46,55,56,60] have demonstrated that a higher F1-score indicates a well-balanced model that does not disproportionately favor either precision or recall. It is defined as:

(4) $$F1-score=2\times \frac{Precision\times Recall}{Precision+Recall}$$

Another important metric is specificity (also called the True Negative Rate), which measures the ability of a model to correctly classify negative cases. Specificity has been reported in several studies [32,36,37,42,45,46,47,54,55] as a key metric for minimizing false positives, ensuring that healthy individuals are not incorrectly classified as having a disease. It is defined as:

(5) $$Specificity=\frac{TN}{TN+FP}$$

Additionally, other evaluation metrics frequently observed in the literature include the confusion matrix and Area Under the Curve (AUC-ROC). The confusion matrix used in studies [40,56,59] provides a detailed performance analysis by displaying the distribution of TP, TN, FP and FN, allowing researchers to interpret misclassification patterns and fine-tune models accordingly. The AUC-ROC metric has been used in several studies [8,27,33,36,44,47,48,52,53,60,64] to evaluate the overall classification ability of a model at various threshold settings, where a higher AUC value indicates better discrimination between diseased and non-diseased cases.

Another robust evaluation metric used in RD classification problems, particularly for imbalanced datasets, is the Matthews Correlation Coefficient (MCC). The MCC metric has been used in a few studies [22,39,46,60,64] to provide a comprehensive measure of a model’s performance by considering all four values in the confusion matrix—TP, TN, FP, and FN. Unlike Acc, MCC is a balanced metric that remains reliable even when the dataset is highly skewed.

In the context of this review, evaluation metrics are presented in a structured manner to enhance clarity and comparison. Although accuracy is widely reported, it is insufficient as a standalone metric, particularly for imbalanced datasets. To ensure a comprehensive evaluation of RD detection models, additional metrics such as recall (sensitivity), specificity, precision, F1-score, and AUC-ROC should be considered. Sensitivity reflects the ability of a model to correctly identify diseased cases and is particularly important in screening tasks, whereas specificity measures the correct identification of healthy cases, thereby reducing false positives. Precision is relevant when minimizing false alarms is critical. The F1-score provides a balance between precision and recall and is suitable for imbalanced data, while AUC-ROC offers a threshold-independent measure for model comparison. However, these metrics are not always directly comparable across studies due to differences in disease prevalence, class distribution, segmentation strategies, and evaluation units. Accordingly, multiple metrics should be used to ensure a comprehensive and context-aware evaluation.

The choice of data modality also influences model performance and applicability. Audio-based approaches, including cough and breath sound analysis, are non-invasive, cost-effective, and suitable for large-scale and remote screening. However, they are sensitive to noise and variability in recording conditions. In contrast, visual-based methods, such as chest X-rays and computed tomography (CT) scans, provide detailed structural information and are more reliable for clinical diagnosis, but require specialized equipment and expertise. In the context of this review, audio-based methods are more appropriate for early detection and scalable screening, whereas visual-based approaches are better suited for diagnostic confirmation. Combining both modalities may further enhance performance and robustness.

## 9. Challenges and Future Directions

This section elaborates on the key challenges in AI-driven RD analysis and discusses future directions to enhance its performance, reliability, and clinical applicability.

### 9.1. Challenges

Despite significant advancements in AI-driven respiratory disease (RD) detection using audio and visual modalities, several challenges remain. One major limitation is the scarcity of coherent multimodal datasets containing synchronized visual data, such as CXR, ultrasound (US), or CT scans, alongside audio data, including cough sounds and breathing patterns, collected from the same patients. This limitation hinders the development of robust multimodal models capable of effectively integrating complementary information from both modalities. In addition, many existing studies rely on relatively small sample sizes and limited high-quality annotated data, particularly for rare respiratory conditions that require expert clinical labeling. Class imbalance also remains a common challenge, as some disease categories are underrepresented compared with normal or more prevalent classes, potentially biasing model performance. Another important concern is the lack of rigorous validation, as several studies evaluate their models using only a single dataset, limiting the generalizability of the reported findings across different populations and clinical settings. Validation on multiple and diverse datasets is therefore essential to ensure model robustness, especially considering variations in data quality, acquisition sources, and patient demographics [92]. Additionally, dataset bias related to demographic imbalance, disease prevalence, or acquisition settings may affect model fairness and robustness, potentially limiting clinical applicability in diverse real-world populations. Furthermore, although many studies have reported promising results on retrospective datasets, such outcomes do not necessarily reflect clinically validated diagnostic or monitoring performance in real-world healthcare environments. Therefore, caution is required when interpreting the reported findings and their potential clinical applicability.

A notable limitation of the current evidence base is the strong dominance of COVID-19-focused studies, particularly in multimodal approaches. While these studies have driven significant progress, they limit the generalizability of findings to other respiratory diseases. The literature is unevenly distributed across conditions, with fewer studies addressing diseases such as asthma and COPD. This highlights the need for more balanced and diverse datasets to support robust and generalizable AI models.

The reviewed studies differ substantially in their evaluation scope, including image-level, recording-level, and patient-level classification tasks, which affects both the interpretation and comparability of reported results. Image-level tasks evaluate individual medical images such as chest X-rays or CT scans, recording-level tasks focus on single audio recordings such as cough, breath, or lung sound samples, while patient-level tasks aggregate information from multiple recordings or images belonging to the same patient. These different evaluation levels influence the complexity of the classification task and the reported model performance. In addition, considerable heterogeneity exists across studies due to differences in disease prevalence, class distribution, dataset size, segmentation strategies, preprocessing methods, and validation protocols. For example, some studies employ balanced datasets and controlled laboratory conditions, whereas others utilize real-world clinical datasets with imbalanced classes and varying data quality. Similarly, validation approaches differ between studies, including hold-out testing, cross-validation, and external validation. This heterogeneity also affects the interpretation of evaluation metrics across studies. While some studies report only accuracy, this metric alone may not provide a comprehensive assessment of model performance, particularly in imbalanced datasets or multi-class classification tasks. Therefore, metrics such as precision, recall, F1-score, specificity, and AUC-ROC should be considered alongside accuracy to provide a more balanced evaluation. Consequently, reported performance metrics should be interpreted within the methodological context of each study and should not be considered directly comparable across different datasets, modalities, and evaluation settings.

Additionally, ablation studies should be conducted to assess the contribution of each model component, providing insights into their individual impact on overall performance. This analysis can help optimize model architecture and guide future enhancements.

Clinical validation and adoption under real-world variability remain key challenges, as most AI models are tested in controlled environments rather than in real-world clinical settings. Without clinical trials and regulatory approvals, integrating these models into healthcare workflows remains a bottleneck [93]. Another concern is interpretability, as many deep learning models function as “black boxes,” making it difficult for clinicians and stakeholders to fully trust their decisions. In addition, regulatory challenges pose barriers to approval and large-scale adoption, with stringent requirements for safety, transparency, and ethical use.

Furthermore, computational requirements are a concern for real-world deployment [65]. Some state-of-the-art AI models are computationally expensive, making them impractical for deployment in resource-limited settings. Lighter and more efficient models should be prioritized for scalability.

Data scarcity, particularly in CT scans and high-quality audio samples, continues to hinder progress [20,94]. The lack of large-scale, high-quality, diverse datasets affects model performance. Additionally, dataset bias and quality issues arise due to variations in data collection methods, sensor noise, and self-reported audio samples, which may not be reliable for clinical applications. Bias in visual data can also arise from variations in imaging protocols and differences in scanner types, impacting image quality and potentially affecting model generalizability and diagnostic accuracy.

Although this review followed the PRISMA methodology, a formal standardized risk-of-bias assessment tool was not applied due to the methodological diversity and technical nature of AI-based studies. Future systematic reviews may benefit from adopting dedicated quality assessment frameworks specifically designed for ML research. Another limitation of this review is that the screening and study selection process was conducted primarily by a single reviewer without independent duplicate screening. Although verification and consistency checks were performed, the possibility of subjective selection bias cannot be entirely excluded.

### 9.2. Future Directions

To address these challenges, future research should focus on enhanced data collection guidelines and strategies [95], ensuring high-quality and well-annotated datasets. Establishing collaborations between IT professionals, AI researchers, and clinicians will be crucial for developing clinically relevant AI solutions. Further, multimodal integration of audio and visual data remains an underexplored area. While some efforts exist, the lack of coherent datasets from single patients limits the reliability of proposed models. Future studies should aim to create synchronized multimodal datasets to improve diagnostic accuracy.

Additionally, explainability and interpretability should be prioritized, as black-box AI models are less likely to gain clinical trust [24]. Explainable models enable clinicians to understand the reasoning behind predictions, thereby supporting informed decision-making and increasing confidence in AI-assisted diagnostics. Techniques such as attention mechanisms, saliency maps, Grad-CAM, and feature importance analysis provide insights into model behavior and help highlight clinically relevant patterns, bridging the gap between AI systems and medical professionals [96]. Furthermore, interpretability is often required for regulatory approval, as healthcare authorities demand transparency to ensure that AI systems operate reliably and without unintended bias.

Furthermore, hybrid AI approaches that integrate DL with traditional signal processing can improve robustness and enhance efficiency. These methods leverage the strengths of both paradigms, allowing for more reliable feature extraction and classification, particularly in complex RD detection tasks.

Finally, efforts should be directed toward regulatory and clinical validation to ensure that AI models meet healthcare standards and are adaptable to real-world clinical environments [97]. Establishing standardized evaluation protocols and compliance with medical regulations will be critical for the safe deployment of AI in clinical practice. Furthermore, collaboration between AI researchers, clinicians, and regulatory bodies will be essential to address these challenges and facilitate the safe and effective integration of AI into healthcare practice [98]. By addressing these gaps, AI-based RD detection using audio-visual-based modalities can advance toward more accurate, efficient, and clinically deployable solutions.

## 10. Conclusions

This review analyzed 45 studies to explore the latest AI-based methodological approaches and their applications in RD detection. It examines three key areas—audio-based, visual-based, and audio-visual modalities—over the past five years. To the best of our knowledge, this is one of the first reviews to systematically address all three domains within a single study, providing a holistic understanding of current advancements in the field. Research in RD detection has been strongly shaped by the COVID-19 pandemic, which shifted much of the focus toward diagnosing COVID-19 using respiratory audio, visual data, or a combination of both, while slightly diverting attention from other respiratory diseases. DL approaches trained directly on raw or high-dimensional features generally outperform traditional ML methods in accuracy, though they require greater computational resources and larger datasets. Ensemble methods improve robustness by combining complementary models but increase system complexity and training cost. These trade-offs highlight that no single approach is universally optimal; lightweight models are better suited for mobile or point-of-care screening, whereas more computationally intensive methods may be appropriate in centralized clinical settings where accuracy is prioritized. A critical gap across many studies is the absence of multimodal datasets, which limits the development of integrated models capable of leveraging both audio- and visual-based signals. Future research should emphasize multimodal integration, combining audio and visual data to enhance diagnostic performance and reliability. The availability of coherent datasets containing both modalities from the same patients will be key to reducing misdiagnosis rates and enabling early detection. Continued progress in multimodal AI models will be essential for developing more accurate, interpretable, and clinically applicable diagnostic solutions, ultimately improving outcomes in RD detection.

## Figures and Tables

**Table 1:** Literature Review Matrix.

Year	Ref	Audio-Based					Visual-Based			Learning Approach			Dataset	Application
		Cough	Breath	Speech	Wheeze	Crackles	CXR	CT	US	FE	Trd. ML	DL		
Audio-Based Studies														
2021	[25]	*	*	*						*		*	O	COVID-19
2022	[22]	*								*	*	*	O	COVID-19
2022	[26]	*								*	*	*	O	Pneumonia
2022	[27]	*	*	*						*	*		O	COVID-19
2022	[28]				*	*				*	*	*	O	Lung diseases
2023	[29]	*								*	*	*	O	Pneumonia
2023	[8]	*								*	*		O	COVID-19
2023	[30]	*	*	*						*	*	*	O	COVID-19
2023	[31]		*							*		*	P	Asthma
2023	[32]	*								*	*	*	P	COVID-19
2023	[33]	*	*							*		*	O	COVID-19
2024	[34]				*	*				*	*	*	O	Asthma
2024	[35]				*	*				*		*	O	Lung diseases
2024	[36]				*	*				*	*		O	Lung diseases
2024	[37]				*	*				*		*	O	Lung diseases
2024	[38]				*	*				*		*	O	COPD
2024	[39]	*	*	*	*	*				*	*	*	O	Lung diseases
2025	[40]				*	*				*	*		O	COPD
Visual-Based Studies														
2020	[41]						*			*		*	O	COVID-19
2020	[23]						*	*	*	*			O	COVID-19
2021	[42]						*			*		*	O	TB
2021	[43]								*			*	O	COVID-19
2022	[44]						*			*		*	O	COVID-19
2022	[45]						*			*	*		O	COVID-19
2022	[46]						*				*	*	O	COVID-19
2022	[47]						*					*	O	TB
2022	[48]						*					*	O	TB
2022	[49]						*					*	O	Pneumonia
2022	[50]							*		*	*		P	TB
2023	[51]						*	*		*	*	*	O	Pneumonia
2023	[52]						*	*				*	O	COVID-19
2023	[53]						*					*	O	Pneumonia
2023	[54]						*					*	O	TB
2024	[55]							*		*	*	*	O	COVID-19
2024	[56]						*					*	O	Pneumonia
2024	[57]						*					*	O	COPD
2024	[58]						*					*	O	TB
2025	[59]						*			*	*	*	O	Pneumonia
Audio-Visual-Based Studies														
2021	[60]	*	*	*			*			*		*	O	COVID-19
2021	[61]		*				*			*		*	O	COVID-19
2022	[62]	*	*	*			*			*	*	*	O	COVID-19
2022	[63]	*	*	*			*			*		*	O & P	COVID-19
2024	[64]	*						*		*	*	*	P	COPD
2024	[65]	*					*			*		*	O	COVID-19
2024	[66]	*	*	*			*			*		*	O & P	COVID-19

Note: FE denotes Feature Engineering; Trd. ML denotes Traditional Machine Learning; DL denotes Deep Learning; O indicates Open dataset; P indicates Private dataset. * indicates the use of the corresponding modality in the study.

**Table 2:** Strengths and Limitations of Open-Source Audio-Based Datasets for RD Detection.

Dataset	Modality Types	Strengths	Limitations
ICBHI [76]	Wheeze, Crackles	Rich source of respiratory sounds, including crackles and wheezes. Event annotations for 10,775 crackles and wheezes aid algorithm development.	Variability in recording conditions; standardization could improve uniformity. Increasing participant number could enhance generalizability.
Coswara [73]	Cough, Speech, Breath	Rich metadata accompanying the audio recordings. Diverse audio modalities.	Inconsistencies in audio quality due to diverse recording environments.
Virufy [71]	Cough	Well-labeled dataset for COVID-19 detection. Collected in a controlled environment.	Small sample size (16 participants) may limit generalizability and model robustness.
The Covid19- Cough [74]	Cough	Remote data collection without geographical constraints.	Variability in audio quality due to differing environments, devices, and user compliance may impact reliability.
NeurIPS [67]	Cough, Breath	Diverse modalities for analysis of COVID-19 biomarkers. Standardized benchmarking enables model comparison on a shared dataset.	Lack of expert annotations. Variability in recording conditions could affect performance.
COUGHVID [72]	Cough	Expert annotations support training and validation. Large number of samples from diverse demographics.	Background noise and uncontrolled recording conditions. Self-reported labels may introduce bias.
KAUH [77]	Wheeze, Crackles	Comprehensive medical annotations. Multiple RD classes provide broad coverage for model training.	Limited sample size (105 subjects) may not capture variability across populations.
Second DiCOVA [75]	Cough, Breath, Speech	Diverse modalities for analysis of COVID-19 biomarkers. Standardized benchmarking enables model comparison.	Crowdsourced data variability may introduce inconsistencies, affecting performance.
Sarcos [78]	Cough	Valuable benchmarking dataset for COVID-19 detection.	Small South Africa dataset may limit generalizability. Recording differences may impact consistency and performance.
SPRSound [79]	Wheeze, Crackles	Strong pediatric focus. Expert annotations enhance reliability.	Single-center data collection may limit broader representativeness.
West China [26]	Cough	Pediatric focus provides a valuable resource for pneumonia detection using audio modality.	Single-center collection may limit diversity. Small sample size (173 participants) may limit generalizability.

**Table 5:** Summary of evaluation metrics commonly used in RD detection.

Metric	Interpretation
Accuracy	Overall correctness of predictions.
Precision	Proportion of predicted positives that are correct.
Recall (Sensitivity)	Proportion of actual positives correctly identified.
Specificity	Proportion of actual negatives correctly identified.
F1-score	Balance between precision and recall.
AUC-ROC	Overall ability to distinguish between classes.

## Data Availability

No new data were created. This literature review is based on previously published sources cited in the manuscript.

## References

[1] World Health Organization (2023). Chronic Respiratory Diseases. https://www.who.int/health-topics/chronic-respiratory-diseases.

[2] World Health Organization (2026). COVID-19 Dashboard: Deaths. https://data.who.int/dashboards/covid19/deaths?n=c.

[3] Forum of International Respiratory Societies (2025). Lung Health Awareness Days. https://firsnet.org/news-events/lung-health-awareness-days/.

[4] World Health Organization (2024). Global Tuberculosis Report. https://iris.who.int/bitstream/handle/10665/379339/9789240101531-eng.pdf?sequence=1.

[5] World Health Organization (2022). Pneumonia: Fact Sheet. https://www.who.int/news-room/fact-sheets/detail/pneumonia.

[6] Sfayyih A.H., Sabry A.H., Jameel S.M., Sulaiman N., Raafat S.M., Humaidi A.J., Kubaiaisi Y.M.A. (2023). Acoustic-based deep learning architectures for lung disease diagnosis: A comprehensive overview. Diagnostics.

[7] Alqudaihi K.S., Aslam N., Khan I.U., Almuhaideb A.M., Alsunaidi S.J., Ibrahim N.M.A.R., Alhaidari F.A., Shaikh F.S., Alsenbel Y.M., Alalharith D.M. (2021). Cough Sound Detection and Diagnosis Using Artificial Intelligence Techniques: Challenges and Opportunities. IEEE Access.

[8] Shati A., Hassan G.M., Datta A. COVID-19 detection system: A comparative analysis of system performance based on acoustic features of cough audio signals. Proceedings of the 2023 IEEE 22nd International Conference on Trust, Security and Privacy in Computing and Communications (TrustCom).

[9] Amose J., Manimegalai P., Priyanga S., Pavithra S., Susmitha B., Ruth S. Wheeze and Crackle Analysis Using Deep Learning. Proceedings of the 2023 7th International Conference on Electronics, Communication and Aerospace Technology (ICECA).

[10] Ali S.W., Rashid M.M., Yousuf M.U., Shams S., Asif M., Rehan M., Ujjan I.D. (2024). Towards the development of the clinical decision support system for the identification of respiration diseases via lung sound classification using 1D-CNN. Sensors.

[11] Al-qaness M.A., Zhu J., AL-Alimi D., Dahou A., Alsamhi S.H., Abd Elaziz M., Ewees A.A. (2024). Chest X-ray Images for Lung Disease Detection Using Deep Learning Techniques: A Comprehensive Survey: MAA Al-qaness et al. Arch. Comput. Methods Eng..

[12] Shati A., Datta A., Mansoor A., Hassan G.M. (2025). ETDHDNet: An advanced DenseNet-based extended texture descriptor for efficient tuberculosis prediction in CXR images. Intell. Based Med..

[13] Kumar S., Kumar H., Kumar G., Singh S.P., Bijalwan A., Diwakar M. (2024). A methodical exploration of imaging modalities from dataset to detection through machine learning paradigms in prominent lung disease diagnosis: A review. Bmc Med. Imaging.

[14] Crowson M.G., Chan T.C. (2020). Machine learning as a catalyst for value-based health care. J. Med. Syst..

[15] Spies N.C., Farnsworth C.W., Wheeler S., McCudden C.R. (2024). Validating, implementing, and monitoring machine learning solutions in the clinical laboratory safely and effectively. Clin. Chem..

[16] Wang X., Peng Y., Lu L., Lu Z., Bagheri M., Summers R.M. Chestx-ray8: Hospital-scale chest x-ray database and benchmarks on weakly-supervised classification and localization of common thorax diseases. Proceedings of the IEEE Conference on Computer Vision and Pattern Recognition.

[17] Rocha B.M., Filos D., Mendes L., Vogiatzis I., Perantoni E., Kaimakamis E., Natsiavas P., Oliveira A., Jácome C., Marques A. (2018). A respiratory sound database for the development of automated classification. Proceedings of the International Conference on Biomedical and Health Informatics (ICBHI).

[18] Kieu S.T.H., Bade A., Hijazi M.H.A., Kolivand H. (2020). A survey of deep learning for lung disease detection on medical images: State-of-the-art, taxonomy, issues and future directions. J. Imaging.

[19] Alahmari S.S., Altazi B., Hwang J., Hawkins S., Salem T. (2022). A comprehensive review of deep learning-based methods for COVID-19 detection using chest X-ray images. IEEE Access.

[20] Kapetanidis P., Kalioras F., Tsakonas C., Tzamalis P., Kontogiannis G., Karamanidou T., Stavropoulos T.G., Nikoletseas S. (2024). Respiratory diseases diagnosis using audio analysis and artificial intelligence: A systematic review. Sensors.

[21] Moher D., Liberati A., Tetzlaff J., Altman D.G., Group T.P. (2009). Preferred Reporting Items for Systematic Reviews and Meta-Analyses: The PRISMA Statement. Ann. Intern. Med..

[22] Ponomarchuk A., Burenko I., Malkin E., Nazarov I., Kokh V., Avetisian M., Zhukov L. (2022). Project Achoo: A practical model and application for COVID-19 detection from recordings of breath, voice, and cough. IEEE J. Sel. Top. Signal Process..

[23] Horry M.J., Chakraborty S., Paul M., Ulhaq A., Pradhan B., Saha M., Shukla N. (2020). COVID-19 Detection Through Transfer Learning Using Multimodal Imaging Data. IEEE Access.

[24] Li X., Zhang L., Yang J., Teng F. (2024). Role of artificial intelligence in medical image analysis: A review of current trends and future directions. J. Med. Biol. Eng..

[25] Lella K.K., Pja A. (2021). Automatic COVID-19 disease diagnosis using 1D convolutional neural network and augmentation with human respiratory sound based on parameters: Cough, breath, and voice. AIMS Public Health.

[26] Liao S., Song C., Wang X., Wang Y. (2022). A classification framework for identifying bronchitis and pneumonia in children based on a small-scale cough sounds dataset. PLoS ONE.

[27] Grant D., McLane I., Rennoll V., West J. (2022). Considerations and challenges for real-world deployment of an acoustic-based COVID-19 screening system. Sensors.

[28] Revathi A., Sasikaladevi N., Arunprasanth D., Amirtharajan R. (2022). Robust respiratory disease classification using breathing sounds (RRDCBS) multiple features and models. Neural Comput. Appl..

[29] Sharan R.V., Qian K., Yamamoto Y. (2023). Automated cough sound analysis for detecting childhood pneumonia. IEEE J. Biomed. Health Inform..

[30] Benmalek E., Elmhamdi J., Jilbab A., Jbari A. (2023). Automatic COVID-19 Detection Using Machine Learning and Voice Recording. Res. Biomed. Eng..

[31] Aptekarev T., Sokolovsky V., Furman E., Kalinina N., Furman G. (2023). Application of Deep Learning for Bronchial Asthma Diagnostics Using Respiratory Sound Recordings. PeerJ Comput. Sci..

[32] Nasab K.A., Mirzaei J., Zali A., Gholizadeh S., Akhlaghdoust M. (2023). Coronavirus diagnosis using cough sounds: Artificial intelligence approaches. Front. Artif. Intell..

[33] Aytekin I., Dalmaz O., Gonc K., Ankishan H., Saritas E.U., Bagci U., Celik H., Çukur T. (2023). COVID-19 detection from respiratory sounds with hierarchical spectrogram transformers. IEEE J. Biomed. Health Inform..

[34] Abadade Y., Benamar N., Bagaa M., Chaoui H. (2024). Empowering healthcare: TinyML for precise lung disease classification. Future Internet.

[35] Alghamdi N.S., Zakariah M., Karamti H. (2024). A deep CNN-based acoustic model for the identification of lung diseases utilizing extracted MFCC features from respiratory sounds. Multimed. Tools Appl..

[36] Babu N., Pruthviraja D., Mathew J. (2024). Enhancing lung acoustic signals classification with eigenvectors-based and traditional augmentation methods. IEEE Access.

[37] Wu C., Ye N., Jiang J. (2024). Classification and Recognition of Lung Sounds Based on Improved Bi-ResNet Model. IEEE Access.

[38] Trung K.L., Anh P.N., Han T.T. (2024). A novel method in COPD diagnosing using respiratory signal generation based on CycleGAN and machine learning. Computer Methods in Biomechanics and Biomedical Engineering.

[39] Karaarslan O., Belcastro K.D., Ergen O. (2024). Respiratory Sound-Based Disease Classification and Characterization With Deep/Machine Learning Techniques. Biomed. Signal Process. Control.

[40] Taloba A.I., Matoog R.T. (2025). Detecting Respiratory Diseases Using Machine Learning-Based Pattern Recognition on Spirometry Data. Alex. Eng. J..

[41] Echtioui A., Zouch W., Ghorbel M., Mhiri C., Hamam H. (2020). Detection Methods of COVID-19. SLAS Technol. Transl. Life Sci. Innov..

[42] Dasanayaka C., Dissanayake M.B. (2021). Deep Learning Methods for Screening Pulmonary Tuberculosis Using Chest X-Rays. Comput. Methods Biomech. Biomed. Eng. Imaging Vis..

[43] Awasthi N., Dayal A., Cenkeramaddi L.R., Yalavarthy P.K. (2021). Mini-COVIDNet: Efficient lightweight deep neural network for ultrasound-based point-of-care detection of COVID-19. IEEE Trans. Ultrason. Ferroelectr. Freq. Control.

[44] Abbasi W.A., Abbas S.A., Andleeb S., Bibi M., Majeed F., Jaleel A., Akhtar M.N. (2022). COVIDX: Computer-aided diagnosis of COVID-19 and its severity prediction with raw digital chest X-ray scans. Quant. Biol..

[45] Patel R.K., Kashyap M. (2022). Automated diagnosis of COVID stages from lung CT images using statistical features in 2-dimensional flexible analytic wavelet transform. Biocybern. Biomed. Eng..

[46] Ragab M., Alshehri S., Alhakamy N.A., Alsaggaf W., Alhadrami H.A., Alyami J. (2022). Machine learning with quantum seagull optimization model for COVID-19 chest X-ray image classification. J. Healthc. Eng..

[47] Urooj S., Suchitra S., Krishnasamy L., Sharma N., Pathak N. (2022). Stochastic Learning-Based Artificial Neural Network Model for an Automatic Tuberculosis Detection System Using Chest X-ray Images. IEEE Access.

[48] Showkatian E., Salehi M., Ghaffari H., Reiazi R., Sadighi N. (2022). Deep learning-based automatic detection of tuberculosis disease in chest X-ray images. Pol. J. Radiol..

[49] Trivedi M., Gupta A. (2022). A lightweight deep learning architecture for the automatic detection of pneumonia using chest X-ray images. Multimed. Tools Appl..

[50] Haq I., Mazhar T., Nasir Q., Razzaq S., Mohsan S.A.H., Alsharif M.H., Alkahtani H.K., Aljarbouh A., Mostafa S.M. (2022). Machine Vision Approach for Diagnosing Tuberculosis (TB) Based on Computerized Tomography (CT) Scan Images. Symmetry.

[51] Bhimavarapu U., Chintalapudi N., Battineni G. (2023). Multi-classification of lung infections using improved stacking convolution neural network. Technologies.

[52] Mukhi S.E., Varshini R.T., Sherley S.E.F. (2023). Diagnosis of COVID-19 from multimodal imaging data using optimized deep learning techniques. SN Comput. Sci..

[53] Arya V., Kumar T. (2023). Enhancing Image for CNN-Based Diagnostic of Pediatric Pneumonia Through Chest Radiographs. Int. J. Adv. Comput. Sci. Appl..

[54] Goswami K.K., Kumar R., Kumar R., Reddy A.J., Goswami S.K. (2023). Deep learning classification of tuberculosis chest X-rays. Cureus.

[55] Dalal S., Singh J.P., Tiwari A.K., Kumar A. (2024). Identification of COVID-19 with CT scans using radiomics and DL-based features. Netw. Model. Anal. Health Inform. Bioinform..

[56] Gowri L., Pradeepa S., Panchada V., Amirtharajan R. (2024). Enhancing pneumonia detection with masked neural networks: A deep learning approach. Neural Comput. Appl..

[57] Karla R., Yalavarthi R. (2024). A hybrid RNN-based deep learning model for lung cancer and COPD detection. Eng. Technol. Appl. Sci. Res..

[58] Chandrasekaran S., Mahesh T.R., Khan S.B., Palaiahnakote S., Alzahrani S. (2024). A hybrid model for classification of tuberculosis chest X-rays images. Malays. J. Comput. Sci..

[59] Shati A., Hassan G.M., Datta A. (2025). A Comprehensive Fusion Model for Improved Pneumonia Prediction Based on KNN-Wavelet-GLCM and a Residual Network. Intell. Syst. Appl..

[60] Jayachitra V.P., Nivetha S., Nivetha R., Harini R. (2021). A cognitive IoT-based framework for effective diagnosis of COVID-19 using multimodal data. Biomed. Signal Process. Control.

[61] Sait U., K.V. G.L., Shivakumar S., Kumar T., Bhaumik R., Prajapati S., Bhalla K., Chakrapani A. (2021). A deep-learning based multimodal system for COVID-19 diagnosis using breathing sounds and chest X-ray images. Appl. Soft Comput..

[62] Kumar S., Chaube M.K., Alsamhi S.H., Gupta S.K., Guizani M., Gravina R., Fortino G. (2022). A novel multimodal fusion framework for early diagnosis and accurate classification of COVID-19 patients using X-ray images and speech signal processing techniques. Comput. Methods Programs Biomed..

[63] Nassif A.B., Shahin I., Bader M., Hassan A., Werghi N. (2022). COVID-19 detection systems using deep-learning algorithms based on speech and image data. Mathematics.

[64] Kumar S., Bhagat V., Sahu P., Chaube M.K., Behera A.K., Guizani M., Gravina R., Dio M.D., Fortino G., Curry E. (2024). A novel multimodal framework for early diagnosis and classification of COPD based on CT scan images and multivariate pulmonary respiratory diseases. Comput. Methods Programs Biomed..

[65] Thandu A.L., Pradeepini G. (2024). Privacy-centric multi-class detection of COVID-19 through breathing sounds and chest X-ray images: Blockchain and optimized neural networks. IEEE Access.

[66] Akhtar F., Mahum R., Ragab A.E., Butt F.S., El-Meligy M.A., Hassan H. (2024). COVID-19 detection systems based on speech and image data using deep learning algorithms. Int. J. Comput. Intell. Syst..

[67] Brown C., Chauhan J., Grammenos A., Han J., Hasthanasombat A., Spathis D., Xia T., Cicuta P., Mascolo C. Exploring automatic diagnosis of COVID-19 from crowdsourced respiratory sound data. Proceedings of the 26th ACM SIGKDD Int. Conf. on Knowledge Discovery and Data Mining (KDD).

[68] Kim S.Y., Lee H.M., Lim C.Y., Kim H.W. (2025). Detection of Abnormal Symptoms Using Acoustic-Spectrogram-Based Deep Learning. Appl. Sci..

[69] Simonyan K., Zisserman A. (2014). Very Deep Convolutional Networks for Large-Scale Image Recognition. arXiv.

[70] Hochreiter S., Schmidhuber J. (1997). Long short-term memory. Neural Comput..

[71] Virufy (2025). Virufy Cough Data Repository. https://github.com/virufy/virufy-data.

[72] Orlandic L., Teijeiro T., Atienza D. (2021). The COUGHVID crowdsourcing dataset, a corpus for the study of large-scale cough analysis algorithms. Sci. Data.

[73] Sharma N., Krishnan P., Kumar R., Ramoji S., Chetupalli S.R., Ghosh P.K., Ganapathy S. (2020). Coswara—A database of breathing, cough, and voice sounds for COVID-19 diagnosis. arXiv.

[74] COVID-19 Cough Dataset Team (2020). COVID-19 Cough Dataset. https://github.com/covid19-cough/dataset.

[75] Sharma N.K., Chetupalli S.R., Bhattacharya D., Dutta D., Mote P., Ganapathy S. (2021). The second DiCOVA challenge: Dataset and performance analysis for COVID-19 diagnosis using acoustics. arXiv.

[76] Rocha B.M., Filos D., Mendes L., Serbes G., Ulukaya S., Kahya Y.P., Jakovljevic N., Turukalo T.L., Vogiatzis I.M., Perantoni E. (2019). An open access database for the evaluation of respiratory sound classification algorithms. Physiol. Meas..

[77] Fraiwan M., Fraiwan L., Khassawneh B., Ibnian A. (2021). A Dataset of Lung Sounds Recorded from the Chest Wall Using an Electronic Stethoscope. Mendeley Data, V3.

[78] Pahar M., Klopper M., Warren R., Niesler T. (2021). COVID-19 cough classification using machine learning and global smartphone recordings. Comput. Biol. Med..

[79] Zhang Q., Zhang J., Yuan J., Huang H., Zhang Y., Zhang B., Lv G., Lin S., Wang N., Liu X. (2022). Sprsound: Open-source SJTU paediatric respiratory sound database. IEEE Trans. Biomed. Circuits Syst..

[80] Soares E., Angelov P., Biaso S., Froes M.H., Abe D.K. (2020). SARS-CoV-2 CT-sCAN Dataset: A Large Dataset of Real Patients CT Scans for SARS-CoV-2 Identification. medRxiv.

[81] Born J., Brändle G., Cossio M., Disdier M., Goulet J., Roulin J., Wiedemann N. (2020). POCOVID-Net: Automatic detection of COVID-19 from a new lung ultrasound imaging dataset (POCUS). arXiv.

[82] Kermany D.S., Goldbaum M., Cai W., Valentim C.C.S., Liang H., Baxter S.L., McKeown A., Yang G., Wu X., Yan F. (2018). Labeled Optical Coherence Tomography (OCT) and Chest X-ray Images for Classification. Mendeley Data, V2.

[83] Jaeger S., Candemir S., Antani S., Wáng Y.X.J., Lu P.X., Thoma G. (2014). Two public chest X-ray datasets for computer-aided screening of pulmonary diseases. Quant. Imaging Med. Surg..

[84] Rahman T., Khandakar A., Kadir M.A., Islam K.R., Islam K.F., Mazhar R., Hamid T., Islam M.T., Kashem S., Mahbub Z.B. (2020). Reliable tuberculosis detection using chest X-ray with deep learning, segmentation and visualization. IEEE Access.

[85] Zhao J., Zhang Y., He X., Xie P. (2020). COVID-CT-Dataset: A CT scan dataset about COVID-19. arXiv.

[86] Rahman T. (2021). COVID-19 Radiography Database. https://www.kaggle.com/datasets/tawsifurrahman/covid19-radiography-database.

[87] Radiological Society of North America (RSNA) (2018). RSNA Pneumonia Detection Challenge 2018. https://www.rsna.org/rsnai/ai-image-challenge/rsna-pneumonia-detection-challenge-2018.

[88] Bustos A., Pertusa A., Salinas J.M., de la Iglesia-Vayá M. (2020). PadChest: A large chest X-ray image dataset with multi-label annotated reports. Med. Image Anal..

[89] Zhao A.C. (2020). COVIDx CXR-2 Dataset. https://www.kaggle.com/datasets/andyczhao/covidx-cxr2.

[90] Cohen J.P., Morrison P., Dao L., Roth K., Duong T.Q., Ghassemi M. (2020). COVID-19 image data collection: Prospective predictions are the future. arXiv.

[91] Talukder M.A. Lung X-Ray Image Dataset. Mendeley Data, V1, 2023. https://data.mendeley.com/datasets/9d55cttn5h/1.

[92] Schwabe D., Becker K., Seyferth M., Klaß A., Schaeffter T. (2024). The METRIC-framework for Assessing Data Quality for Trustworthy AI in Medicine: A Systematic Review. npj Digit. Med..

[93] Topol E.J. (2019). High-performance medicine: The convergence of human and artificial intelligence. Nat. Med..

[94] Das S., Ayus I., Gupta D. (2023). A comprehensive review of COVID-19 detection with machine learning and deep learning techniques. Health Technol..

[95] Ijaz A., Nabeel M., Masood U., Mahmood T., Hashmi M.S., Posokhova I., Rizwan A., Imran A. (2022). Towards using cough for respiratory disease diagnosis by leveraging artificial intelligence: A survey. Inform. Med. Unlocked.

[96] Mohapatra R.K., Jolly L., Dakua S.P. (2025). Advancing explainable AI in healthcare: Necessity, progress, and future directions. Comput. Biol. Chem..

[97] Sfayyih A.H., Sulaiman N., Sabry A.H. (2023). A review on lung disease recognition by acoustic signal analysis with deep learning networks. J. Big Data.

[98] Amann J., Blasimme A., Vayena E., Frey D., Madai V.I., Consortium P. (2020). Explainability for artificial intelligence in healthcare: A multidisciplinary perspective. Bmc Med. Inform. Decis. Mak..

